# Rehabilitation efficacy of neuromuscular training for individuals suffering from knee osteoarthritis: a systematic review and meta-analysis based on randomized controlled trials

**DOI:** 10.3389/fphys.2026.1892963

**Published:** 2026-10-21

**Authors:** Bihui Bai, YuBiao Gu, Li Dingpeng, Xingwen Xie, Fei Yao

**Affiliations:** 1 Shanghai Municipal Hospital of Traditional Chinese Medicine, Shanghai University of Traditional Chinese Medicine, Shanghai, China; 2 Gansu Provincial Hospital of Traditional Chinese Medicine, Gansu, Lanzhou, China; 3 Gansu University of Chinese Medicine, Lanzhou, China; 4 School of Acupuncture-Moxibustion and Tuina, Shanghai University of Traditional Chinese Medicine, Shanghai, China

**Keywords:** knee osteoarthritis, meta-analysis, neuromuscular training, randomized controlled trial, rehabilitation

## Abstract

**Objective:**

This study aimed to comprehensively assess the efficacy of neuromuscular training (NMT) in function, symptoms, quality of life (QoL), pain, and safety in individuals with knee osteoarthritis (KOA), thereby providing evidence-based support for rehabilitation strategies in KOA management.

**Methods:**

This study was conducted in accordance with the Preferred Reporting Items for Systematic Reviews and Meta-Analyses (PRISMA) guidelines. We systematically searched PubMed, Embase, Web of Science, and the Cochrane Library from database inception to August 30, 2025. Randomized controlled trials (RCTs) assessing NMT for KOA were included. For multiple reports originating from the same underlying randomized trial, a trial family was established based on trial registration number, randomized sample size, baseline characteristics, intervention protocols, and follow-up duration to avoid duplicate counting. A random-effects model was applied for meta-analysis, and sensitivity analysis, publication bias assessment, prespecified subgroup analyses, and tests for subgroup interactions were performed. The certainty of evidence for primary outcomes and adverse events was evaluated using the approach proposed by the Grading of Recommendations, Assessment, Development, and Evaluation (GRADE) Working Group.

**Results:**

A total of 17 study reports were included, corresponding to 14 independent RCTs with 1,029 participants, of whom 516 were in the NMT group and 513 in the control group. Compared with control interventions, NMT significantly alleviated KOOS Symptoms (MD = 4.44, 95% *CI*: 1.62, 7.26, *P* = 0.002), KOOS ADLs (MD = 3.94, 95% *CI*: 0.09, 7.79, *P* = 0.04), and KOOS QoL (MD = 5.59, 95% *CI*: 0.97, 10.21, *P* = 0.02). The between-group differences for KOOS Pain (MD = 1.95, 95% *CI*: -0.27, 4.17, *P* = 0.08) and KOOS Sport/Rec (MD = 1.64, 95% *CI* -2.24, 5.53, *P* = 0.41) did not reach statistical significance. The meta-analysis of adverse events showed no statistically significant difference in the incidence of adverse events between the NMT and control groups (RR = 0.83, 95% *CI*: 0.50, 1.37, *P* = 0.46). Nevertheless, given the heterogeneity across studies in the definitions, monitoring, and reporting of adverse events, this result should not be interpreted as evidence of equivalent safety between the interventions. The subgroup analyses suggested differences in certain specific subgroups. Most subgroup interaction tests were not statistically significant; however, a significant interaction was observed for mean age in the KOOS QoL analysis (*P* for interaction = 0.02), which should be interpreted cautiously given the limited number of studies.

**Conclusion:**

NMT may partially alleviate comprehensive symptoms, facilitate activities of daily living, and enhance knee-related QoL in patients with KOA, whereas consistent benefits were not demonstrated for KOOS Pain, sport and recreational function, or most objective functional outcomes. No statistically significant difference in reported adverse events was observed; however, heterogeneity in adverse-event definitions and reporting limits conclusions regarding comparative safety. Additional well-designed RCTs with standardized NMT protocols, adequate sample sizes, and longer follow-up are needed to confirm the magnitude and clinical relevance of these effects.

**Systematic Review Registration:**

https://www.crd.york.ac.uk/PROSPERO/view/CRD420251118258, identifier CRD420251118258.

## 1 Introduction

Knee osteoarthritis (KOA) is a degenerative disorder characterized by subchondral bone remodeling, cartilage degeneration, and chronic synovial inflammation, posing a severe threat to the mobility of middle-aged and older adults (Kolasinski et al., 2020; Gelber, 2024). As population aging accelerates, KOA has become one of the most prevalent chronic conditions worldwide (Cross et al., 2014). The primary clinical manifestations of KOA include knee joint pain and functional limitation, significantly affecting patients’ daily activities (Helito et al., 2023). Given the prolonged treatment course, delayed therapeutic response, and suboptimal outcomes, patients with severe KOA typically experience substantial physical and psychological burdens, accompanied by a marked deterioration in quality of life (QoL). According to projections (Li et al., 2025) on the global and Chinese trends of KOA burden up to 2045, the age-standardized prevalence rate (ASPR) of KOA in China is expected to increase by approximately 2.5%, while the global ASPR of KOA is projected to rise by around 7.8%. Hence, the threat posed by KOA will become even more significant. In addition to active medical treatment, timely and effective rehabilitation interventions are urgently needed to more effectively mitigate the adverse effects of KOA.

In the rehabilitation of KOA, the core goals are pain relief, improvement of joint function, and enhancement of patients’ QoL (Holden et al., 2021). Neuromuscular training (NMT) is an exercise-based therapy that integrates neuromuscular control principles with joint biomechanics. Owing to its therapeutic benefits on function, pain, and QoL in patients with KOA, NMT has gradually drawn increasing attention in both clinical and research settings (Roos et al., 2025). NMT is an exercise intervention model based on the principles of neuromuscular control, joint stability, and motor coordination. Its core objective is not merely to enhance muscle strength, but rather to improve symptoms and functional status of KOA patients by ameliorating motor control strategies, optimizing coordination of periarticular muscle groups, and enhancing dynamic stability. Previous studies have suggested that NMT may positively affect pain, physical function, and QoL in patients with KOA. However, due to considerable heterogeneity across studies in terms of training protocols, intervention dosage, combined treatment modalities, and outcome indicators, the specific benefits and applicable conditions of NMT remain uncertain.

Although recent systematic reviews and meta-analyses have evaluated the effects of NMT on functional outcomes in patients with KOA (Emami et al., 2026), the existing evidence still has certain limitations. Previous studies have primarily focused on overall functional performance, without differentiating treatment responses across different clinical dimensions in individuals with KOA, including key patient-reported outcomes such as symptom alleviation, pain experience, activities of daily living (ADLs), sport and recreational function, and disease-related QoL. Secondly, NMT may exert its effects by improving muscle control and enhancing joint stability; however, its impact on muscle strength, biomechanical parameters, and objective functional performance lacks comprehensive evaluation. Furthermore, previous meta-analyses have paid little systematic attention to the safety of NMT, and have not sufficiently explored whether control type, intervention duration, age, body mass index (BMI), or differences in geographic regions influence the treatment effects of NMT.

Therefore, this study is not merely an update of previous meta-analyses, but rather a further multidimensional assessment of the role of NMT in KOA rehabilitation based on existing evidence. By integrating evidence from randomized controlled trials (RCTs), we systematically analyzed the effects of NMT on symptoms, pain, functional activities, disease-related QoL, safety, muscle strength, biomechanical parameters, and objective functional performance in patients with KOA, and explored potential sources of efficacy differences arising from different intervention modalities, patient characteristics, and study backgrounds through prespecified subgroup analyses. Additionally, subgroup interaction tests were used to evaluate effect differences, and the approach proposed by the Grading of Recommendations, Assessment, Development, and Evaluation (GRADE) Working Group was applied to assess the certainty of evidence for primary outcomes, thereby enhancing the scientific rigor of evidence synthesis and interpretation regarding NMT for the treatment of KOA.

## 2 Methods

This study was conducted in accordance with the Preferred Reporting Items for Systematic Reviews and Meta-Analyses (PRISMA) guidelines (Page et al., 2021). The study protocol was registered with the International Prospective Register of Systematic Reviews (PROSPERO) (CRD420251118258).

### 2.1 Literature search and screening

Two researchers (XWX and FY) independently developed the search strategies, including subject headings and keywords, and searched Web of Science, PubMed, Embase, and Cochrane Library from database inception to August 30, 2025. A comprehensive set of search terms was used, including ‘neuromuscular’, ‘training’, ‘exercise’, ‘sport’, ‘osteoarthritis, knee’, ‘knee osteoarthritides’, ‘osteoarthritis of the knee’, ‘osteoarthritis of knee’, ‘knee osteoarthritis’, and ‘random’. Detailed search strategies are available in **Supplementary Table S1**.

### 2.2 Eligibility criteria

#### 2.2.1 Inclusion criteria

(1) Only published, full-text RCTs were eligible. (2) Participants were individuals diagnosed with knee osteoarthritis according to established clinical and/or radiographic criteria, including the American College of Rheumatology criteria (Altman et al., 1986), regardless of sex, age, or nationality. (3) The intervention effects of NMT on KOA were investigated. (4) The available data were sufficient to calculate the relative risk (RR) or mean difference (MD) and corresponding 95% *confidence interval* (*CI*) for the association of NMT with KOA.

#### 2.2.2 Exclusion criteria

Studies were excluded if they were: (i) duplicate publications of the same data; (ii) reviews, letters, comments, or conference abstracts; (iii) studies lacking sufficient data to calculate RR, MD, and 95% *CI*; or (iv) studies not providing data.

Two researchers (BHB and YBG) independently screened the titles and abstracts of all retrieved records, downloaded the full texts, and assessed each record for eligibility. Any disagreements were reconciled through consensus.

### 2.3 Data extraction

Relevant data were independently extracted by two researchers (DPL and XWX). Any disagreements were reconciled via consensus. A predefined Microsoft Excel spreadsheet was used to collect the following information: publication year, first author, country (study location), registration number, sample size, study design, study population, interventions, intervention duration, control measures, male-to-female ratio, mean age, mean BMI, and adverse events. If continuous data were reported as median with range or median with interquartile range (IQR), the corresponding mean and standard deviation (SD) were estimated using the method proposed by Wan et al. (2014). These estimated values were subsequently used for the meta-analysis.

When data were insufficient, the corresponding authors would be contacted for additional information. When sufficient data were available, they were extracted and transformed as required for the meta-analysis. For multiple reports originating from the same underlying randomized trial, a trial family was established based on trial registration number, randomized sample size, baseline characteristics, intervention protocols, and follow-up duration. When multiple reports from the same trial provided different outcome indicators, they could be used as data sources for the respective outcomes; however, they were not regarded as independent RCT counts. When multiple follow-up time points were available for the same outcome, only one representative time point with relatively complete data reporting was selected for the primary meta-analysis to avoid duplicate counting of the same randomized cohort. Other follow-up time points were retained as supplementary information.

### 2.4 Quality assessment

Two researchers (DPL and XWX) independently assessed the methodological quality of all included RCTs using the Cochrane Handbook for Systematic Reviews of Interventions (version 5.1.0). The assessment covered seven domains: allocation concealment, random sequence generation, blinding of participants and personnel, blinding of outcome assessment, selective reporting, incomplete outcome data, and other sources of bias (Cumpston et al., 2019). Each domain was rated as low risk, unclear risk, or high risk. Risk-of-bias judgments were interpreted on a domain-by-domain basis, and no summary numerical score was assigned to grade the overall methodological quality of individual studies. The certainty of evidence for the primary outcomes was assessed using the approach proposed by the GRADE Working Group, with consideration given to risk of bias, inconsistency, indirectness, imprecision, and publication bias. Quality assessment was performed independently by the two researchers. In case of disagreements, a third experienced investigator (FY) would make the final judgment.

### 2.5 Statistical analysis

All statistical analyses were performed using Review Manager 5.4 and STATA 18.0. For continuous data, the effect size was pooled using the MD with its 95% *CI*. For dichotomous data, the RR along with its 95% *CI* was used for pooling. Heterogeneity was assessed using Cochran’s Q (chi-square test) and the Higgins’*I^2^* statistic. *P* < 0.1 or *I*²>50% was considered indicative of substantial heterogeneity. Meta-analysis was conducted based on a random-effects model with the DerSimonian-Laird estimator. Sensitivity analysis was conducted using the leave-one-out method. Publication bias was evaluated using Egger’s test and funnel plots, with statistical significance set at *P* < 0.05. Additionally, subgroup analyses were conducted based on control type (rehabilitation exercise alone or pharmacotherapy alone), intervention duration (≥12 weeks or <12 weeks), mean age (≥60 years or <60 years), mean BMI (≥28 kg/m² or <28 kg/m²), and geographic region (Asia, Europe, North America, or Oceania). Differences in treatment effects between subgroups were assessed via formal tests for interaction. The *P* values for interaction (*P* for interaction) were reported for all prespecified subgroup comparisons, and statistical significance within an individual subgroup was not considered evidence of a subgroup difference unless supported by the corresponding interaction test. These analyses were conducted to evaluate the stability of the pooled results and to explore potential sources of heterogeneity.

## 3 Results

### 3.1 Literature search and screening

Initially, 892 records were retrieved. After 378 duplicate records were removed, 414 records were excluded based on title and abstract screening. The full texts of the remaining 100 records were reviewed, and 83 records were removed, comprising 45 reviews, 30 studies with non-extractable data, 6 studies with incomplete data, and 2 studies with populations not satisfying the inclusion criteria. Ultimately, 17 study reports from 14 unique RCTs were included (**Figure 1**).

**Figure 1:**
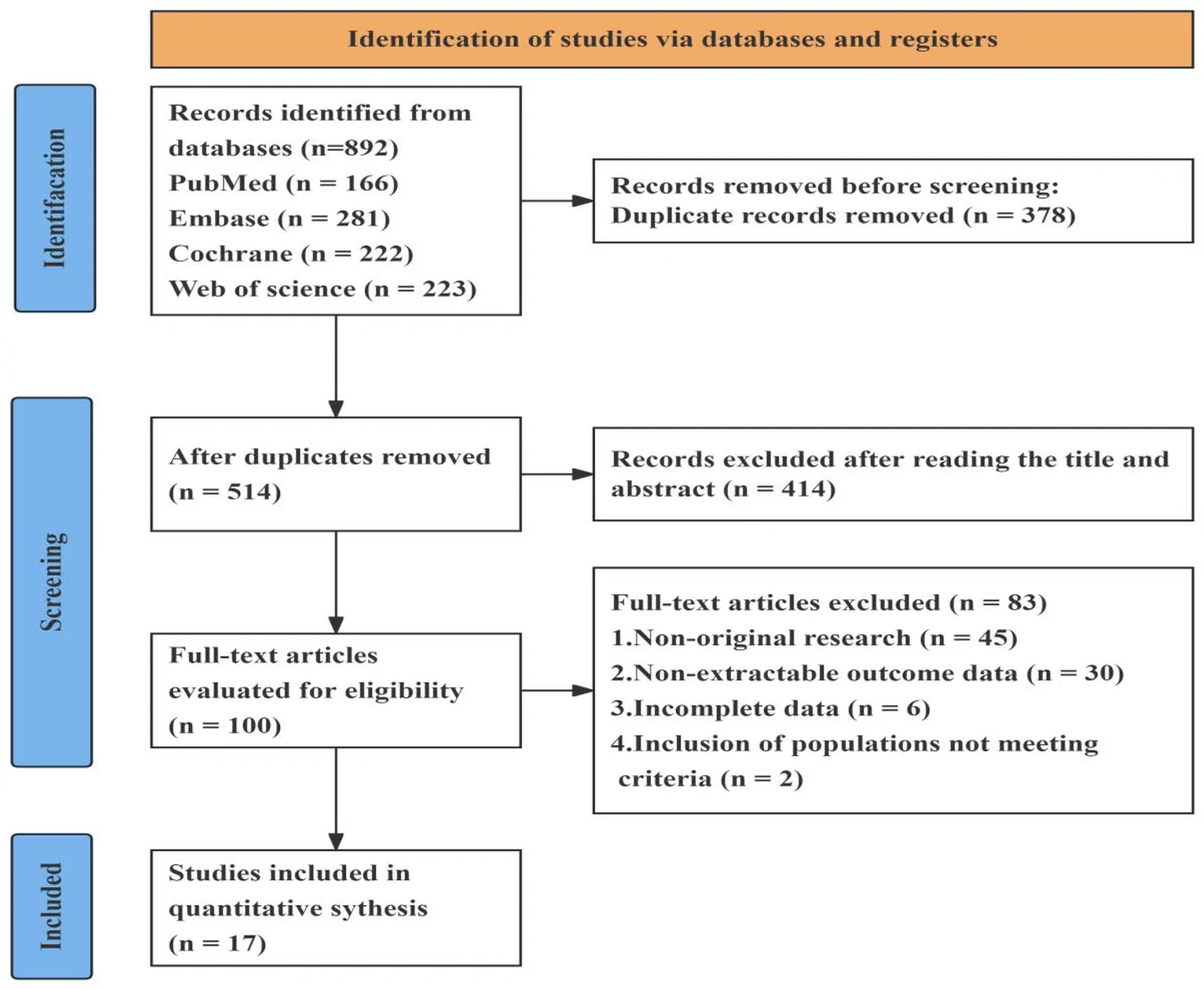
Flow diagram for literature screening.

### 3.2 Characteristics of included reports

The 17 included study reports corresponded to 14 distinct RCTs, with publication years ranging from 2014 to 2025. The included RCTs were conducted in China [Yang 2025 (Yang et al., 2025)], Canada [Lisa 2025 (Carlesso et al., 2025)], India [Sohrab 2023 (Khan et al., 2023), Joshi 2023 (Joshi and Kolke, 2023), Shahzada 2022 (Rashid et al., 2022), Shahzada 2019 (Rashid et al., 2019), Patchava 2017–1 (Apparao et al., 2017a), and Patchava 2017–2 (Apparao et al., 2017b)], Denmark [Holsgaard 2018 (Holsgaard-Larsen et al., 2018), Holsgaard 2017 (Holsgaard-Larsen et al., 2017), Skou 2016 (Skou et al., 2016), Skou 2015 (Skou et al., 2015), and Allan 2014 (Villadsen et al., 2014)], Switzerland [Erika 2015 (Huber et al., 2015)], Norway (Silje 2015 (Stensrud et al., 2015)], and Australia [Michelle 2015 (Hall et al., 2015) and Bennell 2014 (Bennell et al., 2014)]. This study included 17 study reports, which, after verification against a trial family, corresponded to 14 independent RCTs. Some underlying randomized trials generated multiple study reports: Holsgaard 2017 and Holsgaard 2018 both originated from the EXERPHARMA randomized trial (NCT01638962), reporting results at different follow-up time points; Rashid 2019 and Rashid 2022 originated from the same randomized cohort, with Rashid 2022 being a secondary analysis of that randomized clinical trial, primarily supplementing outcomes related to muscle activation; Skou 2015 and Skou 2016 both originated from the MEDIC randomized trial, with Skou 2016 being a pre-specified subsidiary analysis of that trial. Therefore, these homologous study reports were all retained to obtain information at different follow-up time points or on different outcome measures; however, in the counts of independent RCTs and the number of independently randomized participants, they were merged according to the underlying randomized trial. The 14 independent RCTs included a total of 1,029 participants, with 516 in the intervention group and 513 in the control group. Based on the available age data extracted from the table, the mean age was approximately 58.88 years in the intervention group and approximately 58.89 years in the control group. The general characteristics of the included studies are presented in **Table 1**. The raw data extraction results for the 17 included study reports are available in **Supplementary Table S2**. **Supplementary Table S3** presents the trial family correspondence between the study reports and the underlying randomized trials, clarifying the relationship between the 17 study reports and the 14 independent RCTs.

**Table 1:** Basic characteristics of the included literature.

Author	Study period	Region	Study design	Registration number	Population	Intervention	Duration of continuous intervention	Control	Patients (Intervention/Control)	Follow-up
Yang 2025 (Yang et al., 2025)	2014.11–2016.06	China	RCT	ChiCTR-IOR-14005414	KL2–3 KOA	Neuromuscular Exercise + Celecoxib	12 weeks, 3 sessions/week	Celecoxib alone: celecoxib (200 mg/day) with acetaminophen ≤2 g/day allowed; no exercise intervention.	30/29	12 weeks
Lisa 2025 (Carlesso et al., 2025)	2023.04–2024.09	Canada	RCT	NCT05730829	Adults ≥40 with KOA	Neuromuscular Exercise + mind-body + PNE	8 weeks, 2 sessions/week	NEMEX + standard education	35/34	8 weeks
Sohrab 2023 (Khan et al., 2023)	NA	India	RCT	NA	KL2–3 KOA 45-60year	Neuromuscular Training Physiotherapy	4 weeks, 3 sessions/week	Home exercise and general rehabilitation education, with no supervised exercise	30/30	4 weeks
Joshi 2023 (Joshi and Kolke, 2023)	NA	India	RCT	CTRI/2020/11/028949	KL2–3 KOA, 40–65year, NPRS >2–6	Progressive Neuromuscular Training	6 weeks, 2 sessions/week	progressive strength training (ST)	31/31	NA
Shahzada 2022 (Rashid et al., 2022)	2014.01 –2015.02	India	RCT	NA	KOA with varus malalignment (KL≥ 2)	Neuromuscular Training	12 weeks, 3 sessions/week	Quadriceps Training (QT)	31/28	12 weeks
Shahzada 2019 (Rashid et al., 2019)	NA	India	RCT	NA	KOA + varus, KL≥2, VAS≥25 mm	Neuromuscular Training	12 weeks, 3 sessions/week	quadriceps strengthening	31/28	12 weeks
Holsgaard 2018 (Holsgaard-Larsen et al., 2018)	2012.10-2016.05	Denmark	RCT	NCT01638962	KOA, 40-70 year,	Neuromuscular Exercise	8weeks, 2 sessions/week	PHARMA: analgesic/NSAID use	47/46	48 weeks
Holsgaard 2017 (Holsgaard-Larsen et al., 2017)	2012.10–2015.05	Denmark	RCT	NCT01638962	KOA, medial tibiofemoral, KL 0-3, 40-70 year, BMI<32	Neuromuscular Exercise	8 weeks, 2 sessions/week,	PHARMA: instruction on optimal use of acetaminophen & oral NSAIDs (pamphlet+video)	47/46	12 weeks
Patchava 2017-1 (Apparao et al., 2017a)	2015-2017	India	RCT	NA	primary KOA, KL 1-2, ACR criteria, 35-65 year	Neuromuscular Training	8weeks, 3 sessions/week	conventional physical therapy(PT)	45/48	8weeks
Patchava 2017-2 (Apparao et al., 2017b)	NA	India	RCT	NA	primary KOA, KL 1-2, ACR criteria, 40-75 year	Neuromuscular Training	8weeks, 3 sessions/week	Proprioceptive exercises	33/33	NA
Skou 2016 (Skou et al., 2016)	2012.04- 2013.07	Denmark	RCT	NCT01535001	KOA, not eligible for TKR, KL 1–4, 40–75 year, KOOS avg ≤75, VAS ≤60 mm	Neuromuscular Exercise + education (2 sessions) + individualized insoles + dietary weight loss (for BMI ≥ 25) + analgesics as needed.	12 weeks, 2 sessions/week	Two standardized educational booklets	50/50	12 weeks
Skou 2015 (Skou et al., 2015)	2012.04- 2013.07	Denmark	RCT	NCT01535001	KOA (K–L ≥ 1), patients who do not meet the surgical indications for TKR	Neuromuscular exercise + education + individualized insoles ± dietary weight loss ± analgesics.	12weeks, 2 sessions/week	Usual health education	50/50	48 weeks
Erika 2015 (Huber et al., 2015)	2009.05- 2012.06	Switzerland	RCT	NCT00913575	severe KOA, waiting for primary TKR, 55–90 year, able to walk ≥3 m	Neuromuscular Training	4-12weeks	Three preoperative education sessions only, with the same content as the intervention group but without training	22/23	12 weeks
Michelle 2015 (Hall et al., 2015)	2012.05- 2013.11	Australia	RCT	ACTRN12612000542897	30–50 years; 3–12 months post-medial APM with KOA; KOOS pain ≤3/10; KL grade 0–2; BMI ≤36 kg/m²; no other knee surgery	Neuromuscular Training	12weeks	No-intervention control: continued usual daily activities, with no exercise or treatment advice	31/29	13 weeks
Silje 2015 (Stensrud et al., 2015)	2009.10-2012.01	Norway	RCT	NCT01002794	Middle-aged adults (35–60 year) with unilateral symptomatic degenerative meniscus tear, KL ≤2, KOA	Neuromuscular Training	12 weeks 2–3 sessions/week,	Arthroscopic partial meniscectomy (APM)	40/42	12 weeks
Allan 2014 (Villadsen et al., 2014)	2010.01-2011.03	Denmark	RCT	NCT01003756	Severe KOA, scheduled for primary/TKA	Neuromuscular Training+educational package (EP)	8 weeks 2 sessions/week	educational package only (usual-care leaflet)	41/40	13 weeks
Bennell 2014 (Bennell et al., 2014)	2010.07-2012.06	Australia	RCT	ANZCTR12610000660088	Medial KOA + varus malalignment, K/L≥2, VAS≥25 mm	Neuromuscular Training	12 weeks	quadriceps strengthening	50/50	13 weeks

NA, not available.

### 3.3 Quality assessment

A detailed quality assessment is provided in **Figure 2**. **Supplementary Table S4** details the quality assessment of all included RCTs. The certainty of evidence for the primary outcomes and adverse events was evaluated using the approach proposed by the GRADE Working Group, with the specific results presented in **Supplementary Table S5**.

**Figure 2:**
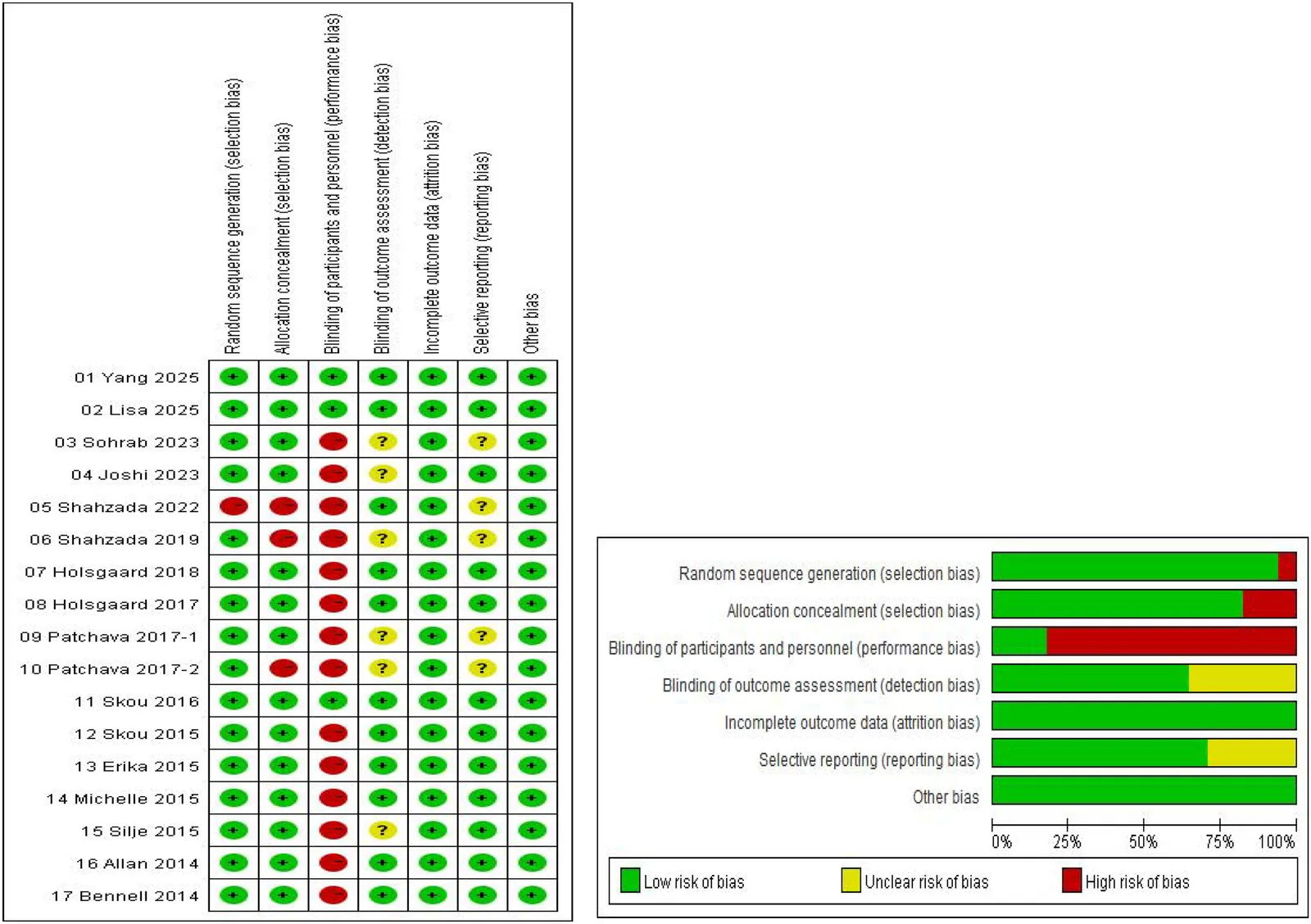
Quality assessment.

### 3.4 Results of primary outcomes from the meta-analysis

#### 3.4.1 Intervention effect on Knee injury and Osteoarthritis Outcome Score symptoms

KOOS Symptoms mainly reflect the comprehensive knee-related symptoms of patients with KOA, including knee stiffness, swelling, activity limitation, and mechanical discomfort. A higher score indicates more effective symptom control. Eight studies reported KOOS Symptoms. The meta-analysis revealed that the NMT group outperformed the control group in improving KOOS Symptoms, with a statistically significant difference (MD = 4.44, 95% *CI*: 1.62, 7.26, *P* = 0.002) (**Figure 3**). Therefore, these results suggest that NMT may alleviate comprehensive knee symptoms, with low heterogeneity (*I²* = 23%).

**Figure 3:**
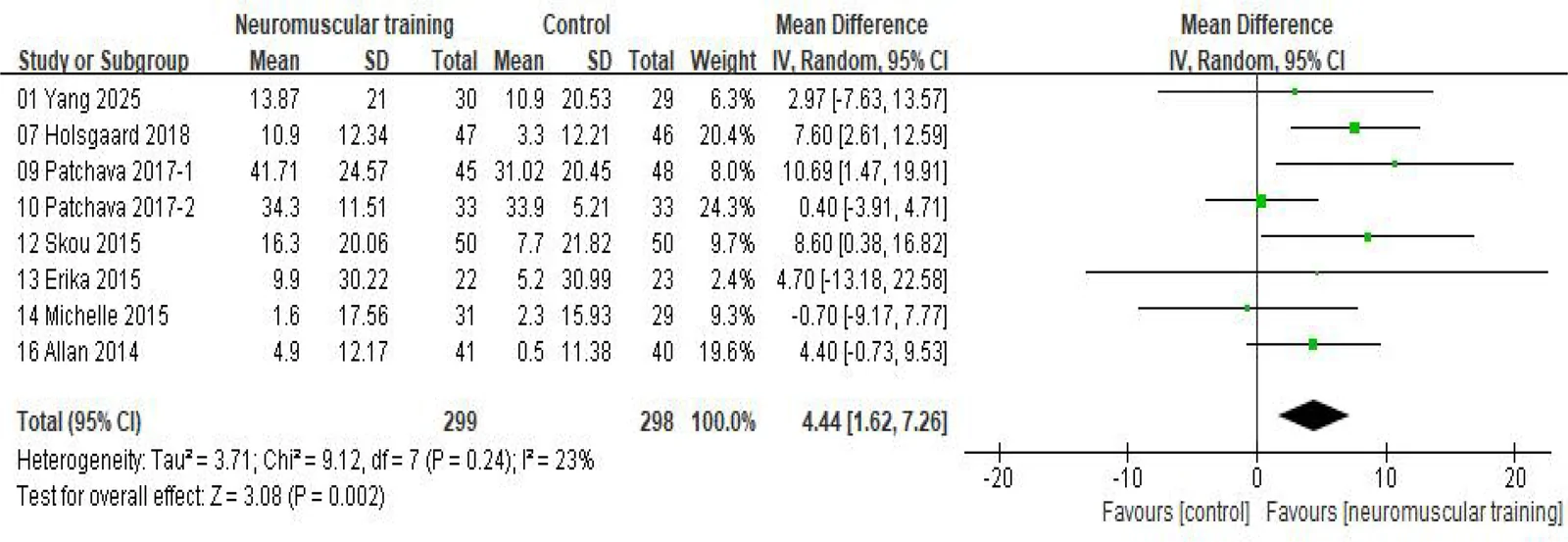
Forest plot for the continuous variable KOOS symptoms.

#### 3.4.2 Intervention effect on KOOS pain and sensitivity analysis

KOOS Pain primarily assesses the pain experience of individuals with KOA during walking, stair climbing, night rest, and weight-bearing activities. A higher score indicates a more favorable pain-related status. Nine studies reported KOOS Pain. Because both randomized groups in Lisa 2025 received NEMEX, this study was excluded from the primary pooled analysis and evaluated separately in a sensitivity analysis. The primary meta-analysis of the remaining eight studies indicated that the difference between the NMT group and the control group in mitigating KOOS Pain did not achieve statistical significance (MD = 1.95, 95% *CI*: -0.27, 4.17, *P* = 0.08, *I*²=0%) (**Figure 4A**). When Lisa 2025 was included in the sensitivity analysis, the pooled effect remained non-significant (MD = 1.27, 95% *CI*: -1.10, 3.63, *P* = 0.29, *I*²=15%), and the overall interpretation remained unchanged (**Figure 4B**).

**Figure 4:**
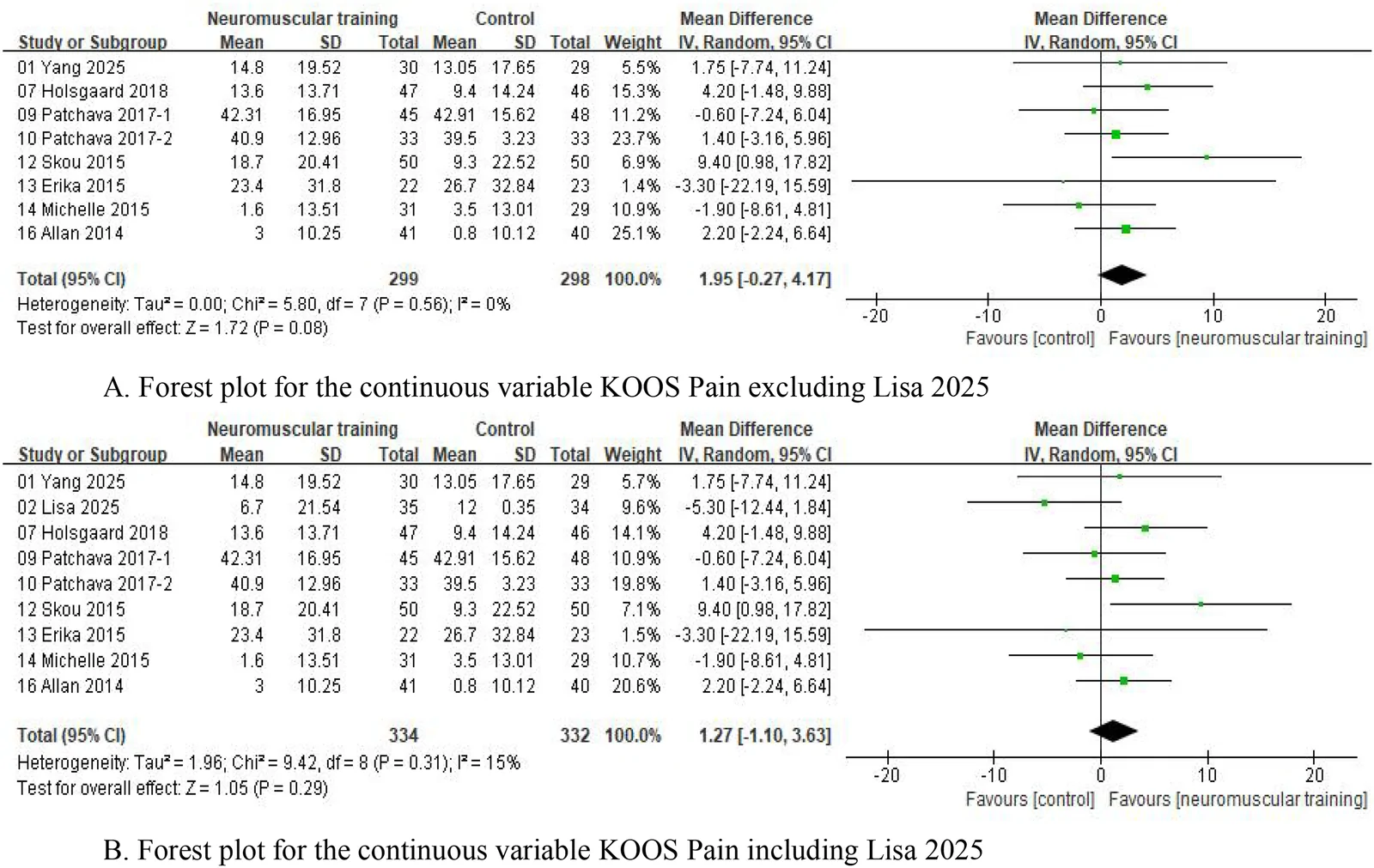
**(A)** Forest plot for the continuous variable KOOS Pain excluding Lisa 2025. **(B)** Forest plot for the continuous variable KOOS Pain including Lisa 2025.

#### 3.4.3 Intervention effect on KOOS ADLs and sensitivity analysis

KOOS ADLs are used to assess the ability of patients with KOA to perform ADLs, including standing up, walking, turning, stair climbing, and squatting. A higher score represents superior basic functional performance in daily life. Seven studies reported KOOS ADLs. The difference in KOOS ADLs between the NMT and control groups was statistically significant (MD = 3.94, 95% *CI*: 0.09, 7.79, *P* = 0.04, *I*²=54%) (**Figure 5**). In an additional sensitivity analysis excluding Patchava 2017-2, the pooled effect remained statistically significant (MD = 5.44, 95% *CI*: 2.39, 8.48, P = 0.0005, *I²* = 5%), indicating that the direction of the overall finding was robust.

**Figure 5:**
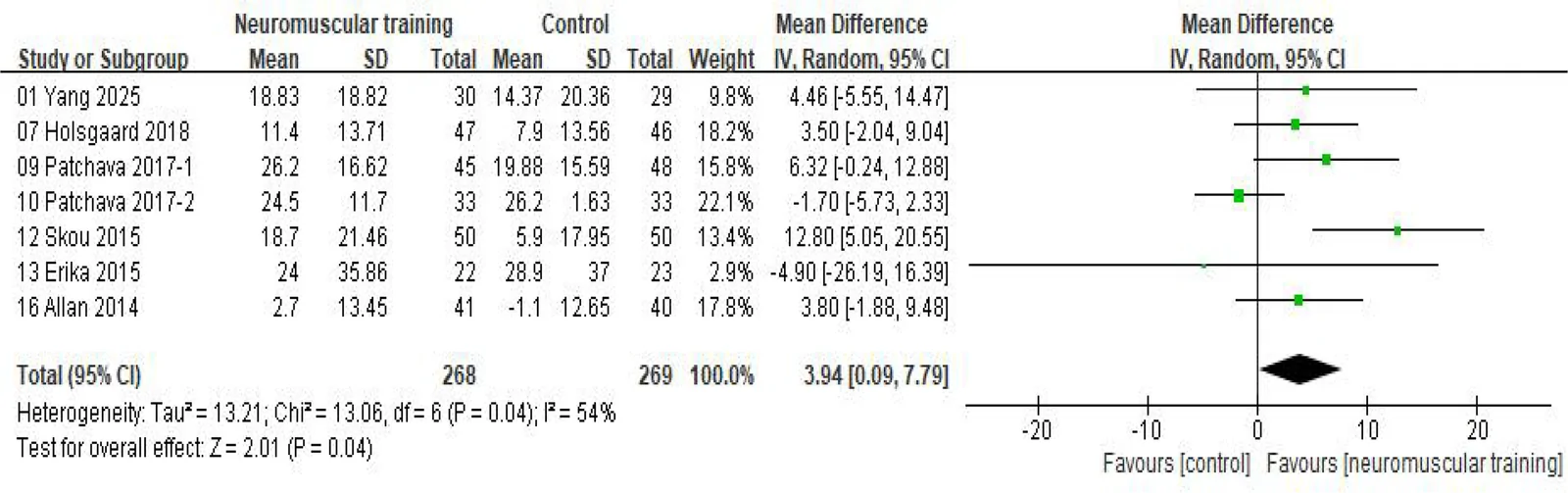
Forest plot for the continuous variable KOOS ADLs.

#### 3.4.4 Intervention effect on KOOS sport and recreation (Sport/Rec)

KOOS Sport/Rec mainly assesses higher-level functional performance, including running, jumping, rapid turning, and participation in sports and recreational activities. A higher score indicates greater ability to perform high-level activities. Data on KOOS Sport/Rec were provided in six studies. The meta-analysis demonstrated that the difference between the NMT group and the control group in KOOS Sport/Rec did not achieve statistical significance (MD = 1.64, 95% *CI* -2.24, 5.53, *P* = 0.41, *I*² =0%) (**Figure 6**).

**Figure 6:**
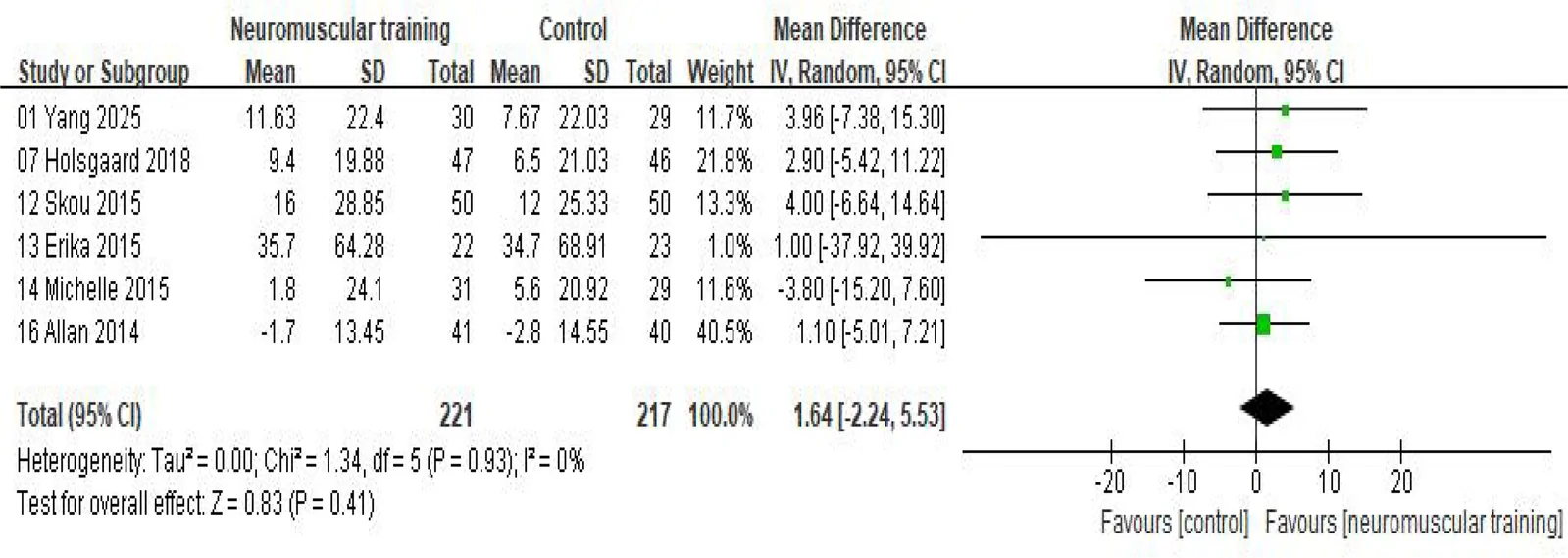
Forest plot for the continuous variable KOOS Sport/Rec.

#### 3.4.5 Intervention effect on KOOS QoL and sensitivity analysis

KOOS QoL reflects patients’ subjective perception of the burden of knee joint disease, including concerns about knee status, worries about future function, life troubles caused by knee joint disease, and self-perceived QoL. Eight studies were included in the primary analysis of KOOS QoL. Lisa 2025 was excluded from the primary pooled analysis because both randomized groups received NEMEX; therefore, this trial represented a comparison between two NEMEX-based intervention strategies rather than a conventional NMT-versus-control contrast. The primary meta-analysis showed that the NMT group outperformed the control group in improving KOOS QoL, with a statistically significant difference (MD = 5.59, 95% *CI*: 0.97, 10.21, *P* = 0.02, *I²* = 71%) (**Figure 7A**). When Lisa 2025 was included in an additional sensitivity analysis, the pooled effect remained statistically significant (MD = 5.31, 95% *CI*: 1.07, 9.55, *P* = 0.01, *I²* = 67%), without materially altering the direction or statistical interpretation of the result (**Figure 7B**).

**Figure 7:**
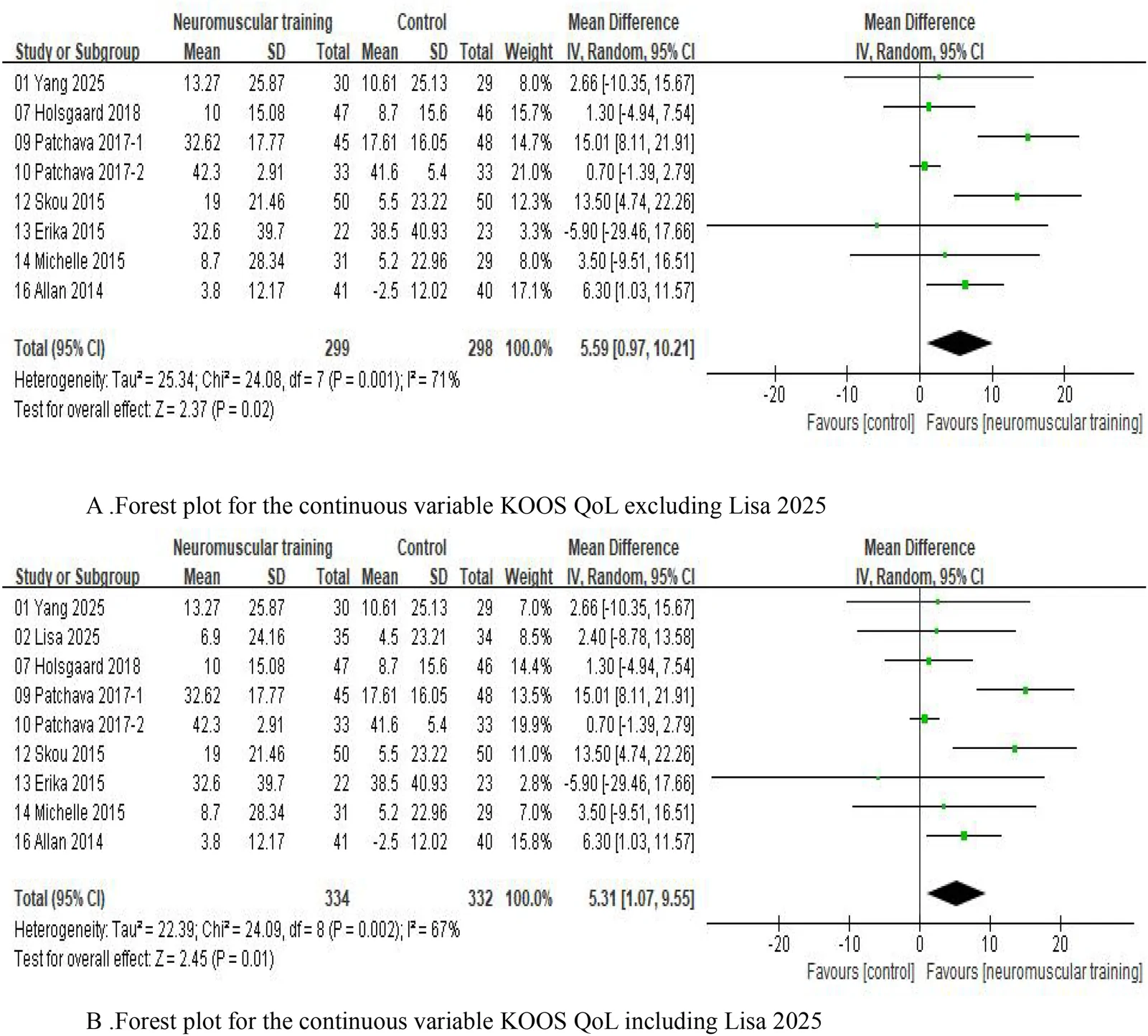
**(A)** Forest plot for the continuous variable KOOS QoL excluding Lisa 2025. **(B)** Forest plot for the continuous variable KOOS QoL including Lisa 2025.

#### 3.4.6 Effect of NMT on adverse events among individuals with KOA and sensitivity analysis

A total of 10 independent RCTs were included. Based on the available evidence, we did not observe a statistically significant difference in the reporting of adverse events between the NMT group and the control group (RR = 0.83, 95% *CI*: 0.50, 1.37, *P* = 0.46, *I²* = 0%) (**Figure 8A**). Given that the definitions, monitoring, and reporting methods for adverse events varied across studies, this result should not be interpreted as equivalence in safety between the two interventions, nor can it completely rule out potential harm. Sensitivity analysis revealed that the direction or statistical interpretation of the pooled estimate was not materially altered (**Figure 8B**). Publication bias was assessed through a funnel plot and Egger’s test. The funnel plot was overall symmetrical. Nevertheless, Egger’s test suggested that publication bias or small-sample study effects possibly existed (Egger: p=0.01) (**Figure 8C**).

**Figure 8:**
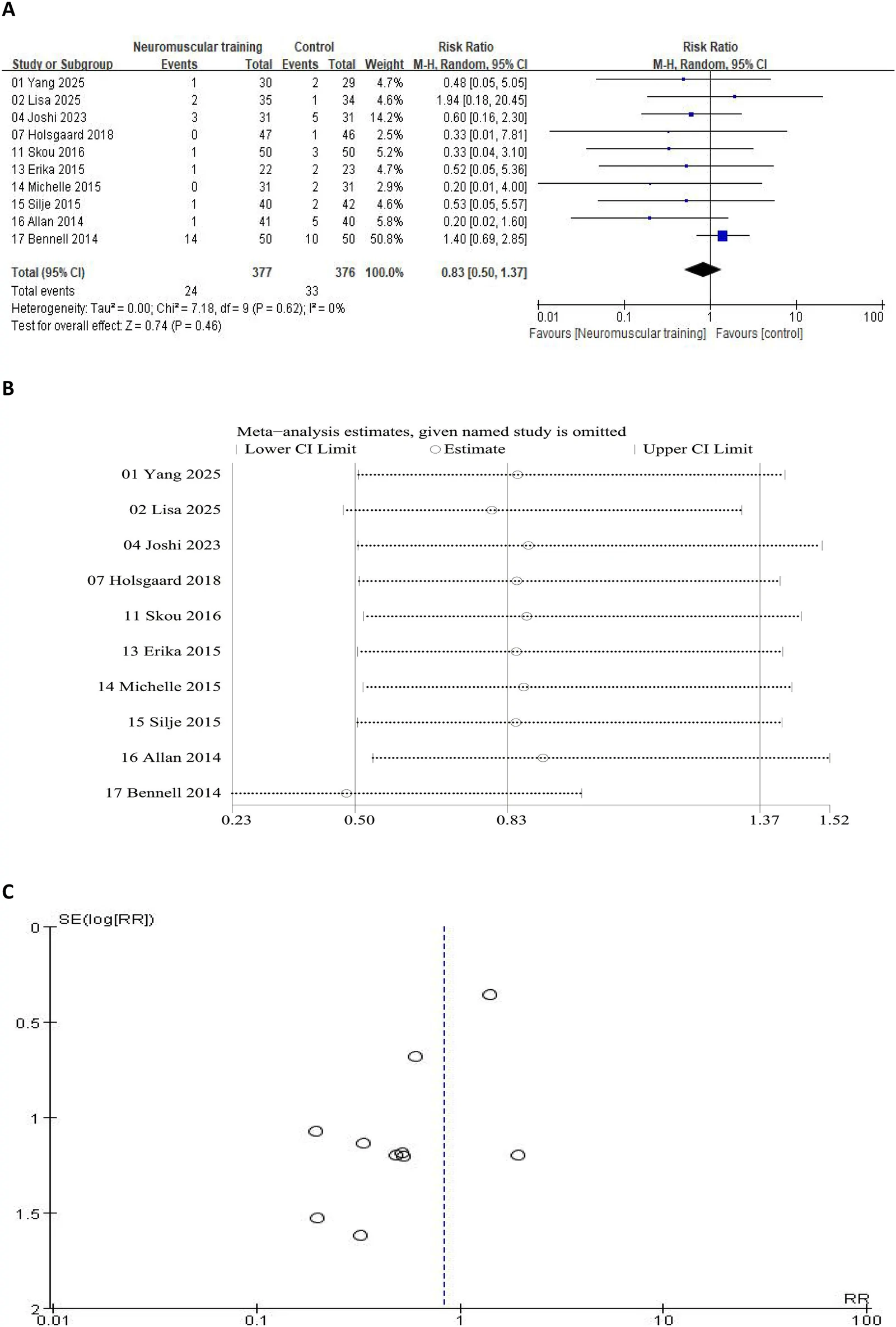
**(A)** Forest plot for the categorical variable adverse events. **(B)** Sensitivity analysis for the categorical variable adverse events. **(C)** Funnel plot for the categorical variable adverse events.

### 3.5 Subgroup analysis

To further explore potential influencing factors, pre-specified subgroup analyses were conducted for the continuous variables of KOOS Symptoms, KOOS Pain, KOOS ADLs, KOOS Sport/Rec, and KOOS QoL. Stratified analyses were performed according to control type (rehabilitation exercise alone or pharmacotherapy alone), intervention duration (≥12 weeks or <12 weeks), mean age (≥60 years or <60 years), mean BMI (≥28 kg/m² or <28 kg/m²), and geographic region (Asia, Europe, North America, or Oceania), and interaction tests between subgroups were performed to evaluate differences in effects across subgroups. For the exploratory subgroup analyses, all eligible NMT-related randomized comparisons, including Lisa 2025, were retained to examine whether study-level characteristics were associated with differences in treatment effects across the broader body of evidence. Detailed results for subgroup analyses and interaction tests are presented in **Supplementary Table S6**; **Tables 2**, **3**.

**Table 2:** Subgroup analysis table for changes in KOOS Symptoms, KOOS Pain, and KOOS ADL as continuous variables.

Subgroup	Change in KOOS symptoms				Change in KOOS pain				Change in KOOS ADL			
	Study	MD [95%CI]	P value (Z-test)	*I^2^*	Study	MD [95%CI]	P value (Z-test)	*I^2^*	Study	MD [95%CI]	P value (Z-test)	*I^2^*
Total	8	4.44 [1.62, 7.26]	0.002	23%	9	1.27 [-1.10, 3.63]	0.29	15%	7	3.94 [0.09, 7.79]	0.04	54%
Control group												
Rehabilitation exercise alone	6	3.77 [0.32, 7.21]	0.03	27%	7	0.67 [-2.23, 3.57]	0.65	27%	5	4.14 [-1.43, 9.71]	0.15	69%
Drug therapy alone	2	4.44 [1.62, 7.26]	0.002	0%	3	3.55 [-1.32, 8.43]	0.15	0%	2	3.73 [-1.12, 8.57]	0.13	0%
Test for subgroup differences			0.3	6.20%			0.32	0%			0.91	0%
Intervention duration												
≥12week	4	3.90 [-1.06, 8.85]	0.12	0%	4	2.19 [-3.66, 8.03]	0.46	34%	3	7.29 [-1.12, 15.70]	0.09	40%
<12week	4	4.93 [0.81, 9.05]	0.02	55	5	1.03 [-1.63, 3.68]	0.45	16%	4	2.42 [-1.22, 6.06]	0.19	46%
Test for subgroup differences			0.75	0%			0.72	0%			0.3	7.70%
ean/median Age												
≥60year	4	5.18 [1.25, 9.10]	0.01	0%	5	1.45 [-3.41, 6.31]	0.56	45%	4	6.12 [0.52, 11.73]	0.03	35%
<60year	3	5.97 [1.74, 10.21]	0.006	36%	3	1.53 [-1.61, 4.67]	0.34	0%	2	0.54 [-4.51, 5.58]	0.83	55%
Test for subgroup differences			0.79	0%			0.98	0%			0.15	52.50%
Mean BMI(kg/m²)												
≥28	3	5.53 [1.30, 9.76]	0.01	0%	4	1.36 [-4.83, 7.56]	0.67	58%	3	6.39 [-1.63, 14.41]	0.12	56%
<28	3	4.38 [-0.88, 9.64]	0.1	31%	3	1.67 [-2.28, 5.61]	0.41	0%	3	3.73 [-1.12, 8.57]	0.13	0%
Test for subgroup differences			0.74	0%			0.93	0%			0.58	0%
Region												
Asia	3	3.77 [-2.48, 10.03]	0.24	49%	3	0.89 [-2.60, 4.39]	0.62	0%	3	2.27 [-3.48, 8.02]	0.44	58%
Europe	4	6.39 [3.17, 9.62]	0.0001	0%	4	3.70 [0.52, 6.88]	0.02	0%	4	5.50 [0.52, 10.48]	0.03	42%
North America	NA	NA	NA	NA	1	-5.30 [-12.44, 1.84]	0.15	NA	NA	NA	NA	NA
Oceania	1	-0.70 [-9.17, 7.77]	0.87	NA	1	-1.90 [-8.61, 4.81]	0.58	NA	NA	NA	NA	NA
Test for subgroup differences			0.27	22.70%			0.09	51.30%			0.41	0%

MD, mean difference; 95% CI, corresponding 95% confidence interval; *I²*, I-squared statistic.

**Table 3:** Subgroup analysis table for changes in KOOS Sport/Rec, KOOS QoL as continuous variables.

Subgroup	Change in KOOS Sport/Rec				Change in KOOS QoL			
	Study	MD [95%CI]	P value (Z-test)	*I^2^*	Study	MD [95%CI]	P value (Z-test)	*I^2^*
Total	6	1.64 [-2.24, 5.53]	0.41	0%	9	5.31 [1.07, 9.55]	0.01	67%
Control group								
Rehabilitation exercise alone	4	0.82 [-3.94, 5.59]	0.73	0%	7	4.42 [-0.07, 8.91]	0.05	71%
Drug therapy alone	2	3.27 [-3.44, 9.98]	0.34	0%	2	9.19 [-1.21, 19.59]	0.08	45%
Test for subgroup differences			0.56	0%			0.41	0%
Intervention duration								
≥12week	4	1.50 [-4.82, 7.83]	0.64	0%	4	6.65 [-0.75, 14.04]	0.08	24%
<12week	3	1.73 [-3.19, 6.65]	0.49	0%	5	4.94 [-0.18, 10.06]	0.06	77%
Test for subgroup differences			0.96	0%			0.71	0%
ean/median Age								
≥60year	4	2.18 [-2.58, 6.95]	0.37	0%	5	6.54 [2.22, 10.86]	0.003	8%
<60year	2	0.57 [-6.15, 7.29]	0.87	0%	3	0.82 [-1.14, 2.78]	0.41	0%
Test for subgroup differences			0.7	0%			0.02	82.10%
Mean BMI(kg/m²)								
≥28	3	1.80 [-3.44, 7.05]	0.5	0%	4	6.91 [1.56, 12.27]	0.01	25%
<28	3	1.45 [-4.33, 7.23]	0.62	0%	3	1.44 [-2.74, 5.62]	0.5	0%
Test for subgroup differences			0.93	0%			0.11	59.90%
Region								
Asia	1	3.96 [-7.38, 15.30]	0.49	NA	3	6.16 [-4.30, 16.62]	0.25	87%
Europe	4	2.12 [-2.32, 6.55]	0.35	0%	4	5.72 [-0.02, 11.45]	0.05	49%
North America	NA	NA	NA	NA	1	2.40 [-8.78, 13.58]	0.67	NA
Oceania	1	-3.80 [-15.20, 7.60]	0.51	NA	1	3.50 [-9.51, 16.51]	0.6	NA
Test for subgroup differences			0.58	0%			0.95	0%

MD, mean difference; 95% CI, corresponding 95% confidence interval; *I²*, I-squared statistic.

#### 3.5.1 Subgroup analysis of KOOS symptoms

For KOOS Symptoms, the overall meta-analysis showed that NMT significantly alleviated symptoms (MD = 4.44, 95% *CI*: 1.62, 7.26, *P* = 0.002, *I^2^ =* 23%). Subgroup analyses showed that symptoms were alleviated in the ‘rehabilitation exercise alone’ subgroup, the ‘pharmacotherapy alone’ subgroup, the ‘mean age ≥60 years’ subgroup, the ‘mean age <60 years’ subgroup, the ‘BMI ≥28 kg/m²’ subgroup, and the ‘Europe’ subgroup (*P*<0.05). However, no statistically significant subgroup interactions were identified for control type (*P* for interaction=0.30), intervention duration (*P* for interaction=0.75), mean age (*P* for interaction=0.79), mean BMI (*P* for interaction=0.74), or geographic region (*P* for interaction=0.27). Therefore, these factors could not be confirmed as effect modifiers of NMT for KOOS Symptoms.

#### 3.5.2 Subgroup analysis of KOOS pain

For the exploratory subgroup analysis of KOOS Pain, in which all eligible NMT-related randomized comparisons including Lisa 2025 were retained, the pooled estimate was not statistically significant (*P* = 0.29, *I^2^ =* 15%); however, alleviation was observed in the ‘Europe’ subgroup (MD = 3.70, 95% *CI*: 0.52, 6.88, *P* = 0.02, *I^2^ =* 0%). No statistically significant subgroup interactions were identified for control type (*P* for interaction = 0.32), intervention duration (*P* for interaction = 0.72), mean age (*P* for interaction = 0.98), mean BMI (*P* for interaction = 0.93), or geographic region (*P* for interaction = 0.09). Therefore, the observed effect within the European subgroup should be interpreted with caution and should not be considered evidence of a definitive regional difference.

#### 3.5.3 Subgroup analysis of KOOS ADLs

For KOOS ADLs, the overall analysis showed that NMT enhanced patients’ ability to perform ADLs (MD = 3.94, 95% *CI*: 0.09, 7.79, *P* = 0.04, *I^2^ =* 54%). After further stratification, improvement was observed in the ‘mean age ≥60 years’subgroup (MD = 6.12, 95% *CI*: 0.52, 11.73, *P* = 0.03, *I^2^ =* 35%). However, no statistically significant subgroup interactions were identified for control type (*P* for interaction = 0.91), intervention duration (*P* for interaction = 0.30), mean age (*P* for interaction = 0.15), mean BMI (*P* for interaction = 0.58), or geographic region (*P* for interaction = 0.41). Given the moderate overall heterogeneity (*I^2^* = 54%) and the absence of statistically significant interaction tests, none of the prespecified subgroup variables could be confirmed as explaining the observed between-study heterogeneity in KOOS ADLs.

#### 3.5.4 Subgroup analysis of KOOS Sport/Rec

For KOOS Sport/Rec, neither the overall analysis nor any of the pre-specified subgroup analyses indicated statistically significant improvements (*P*>0.05, *I^2^ =* 0%), suggesting that evidence for the effect of NMT on the recovery of higher-level motor function remained limited. No statistically significant subgroup interactions were identified for control type (*P* for interaction = 0.56), intervention duration (*P* for interaction = 0.96), mean age (*P* for interaction = 0.70), mean BMI (*P* for interaction = 0.93), or geographic region (*P* for interaction = 0.58).

#### 3.5.5 Subgroup analysis of KOOS QoL

For the exploratory subgroup analysis of KOOS QoL, which retained Lisa 2025, the pooled effect was statistically significant (MD = 5.31, 95% *CI:* 1.07, 9.55, *P* = 0.01, *I^2^ =* 67%). Subgroup analyses showed improvements in the’mean age ≥60 years’subgroup (MD = 6.54, 95% *CI*: 2.22, 10.86, *P* = 0.003, *I^2^ =* 8%) and the ‘BMI ≥28 kg/m²’subgroup (MD = 6.91, 95% *CI*: 1.56, 12.27, *P* = 0.01, *I^2^ =* 25%). The interaction test for mean age was statistically significant (*P* for interaction = 0.02), suggesting a potential age-related difference in the effect of NMT on KOOS QoL. In contrast, no statistically significant subgroup interactions were identified for control type (*P* for interaction = 0.41), intervention duration (*P* for interaction = 0.71), mean BMI (*P* for interaction = 0.11), or geographic region (*P* for interaction = 0.95). Among the prespecified subgroup variables, only mean age showed a statistically significant interaction, suggesting that age may account for some of the observed between-study variation in KOOS QoL. However, given the limited number of studies within the subgroups and the use of study-level mean age rather than individual participant data, this finding should be interpreted cautiously, regarded as exploratory, and should not be considered a complete explanation for the observed heterogeneity.

Overall, except for the interaction between mean age and KOOS QoL (*P* for interaction = 0.02), none of the formal interaction tests provided statistically significant evidence that the prespecified subgroup variables modified the effects of NMT. Therefore, statistically significant effects observed within individual subgroups, including those defined by BMI and geographic region, should be interpreted as exploratory findings rather than evidence of definitive subgroup-specific treatment effects. For outcomes with moderate to high heterogeneity, the prespecified subgroup variables did not fully account for the observed between-study heterogeneity.

### 3.6 Results of secondary outcomes from the meta-analysis

#### 3.6.1 Analysis of pain-related secondary outcomes

Among pain-related secondary outcomes, the pooled analyses of VAS pain intensity after walking and overall VAS pain showed no statistically significant between-group differences (*P*>0.05) (**Figures 9A, B**). Overall, the additional evidence for pain relief with NMT was not sufficient.

**Figure 9:**
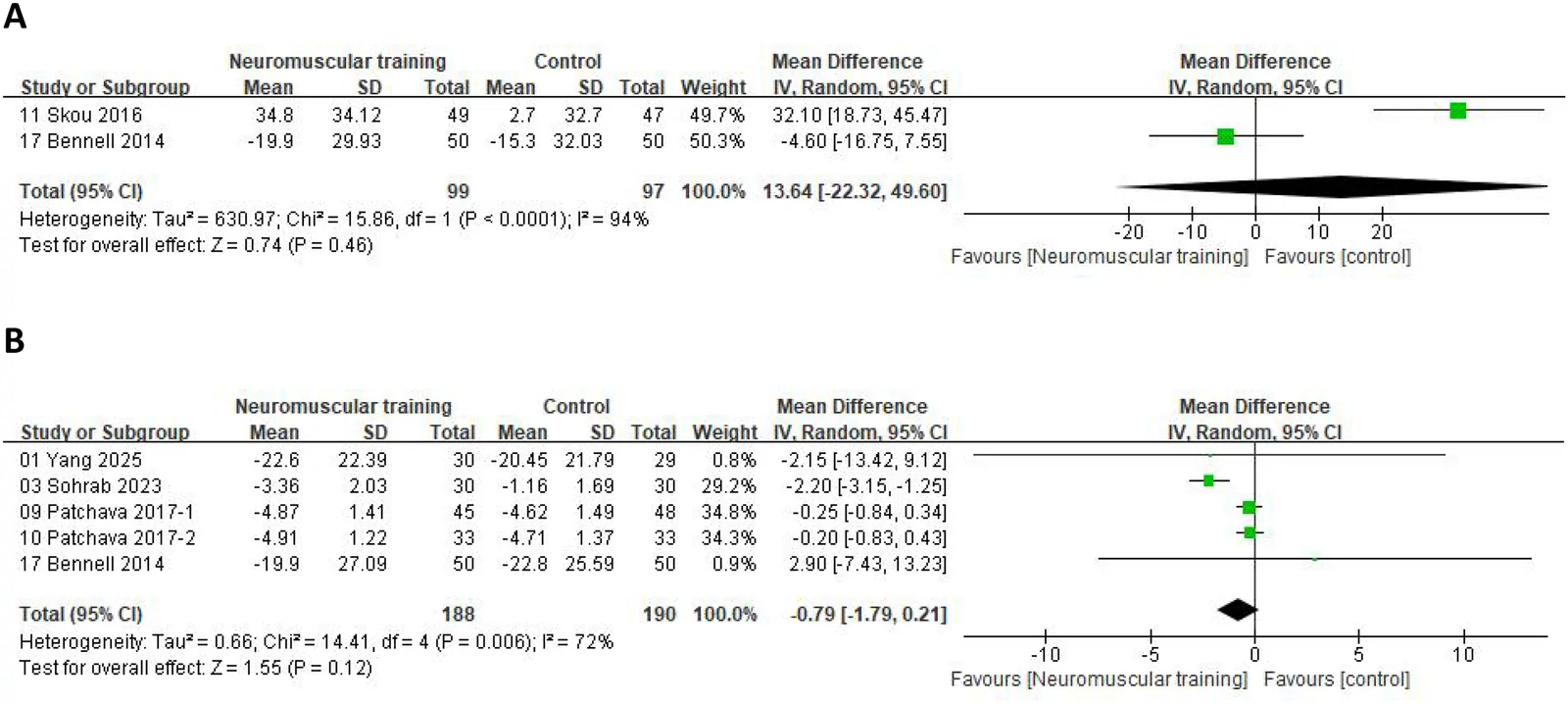
**(A)** Forest plot for the continuous variable VAS Pain intensity after walking. **(B)** Forest plot for the continuous variable VAS.

#### 3.6.2 Analysis of QoL-related secondary outcomes

In terms of QoL indicators related to general health, analyses of EQ-5D-3L VAS and EQ-5D-3L showed no statistically significant differences (*P*>0.05) (**Figures 10A, B**). This indicated that, aside from the KOOS-specific knee-related QoL dimension, evidence for improvement in broader health status and self-rated health perception with NMT remained unclear.

**Figure 10:**
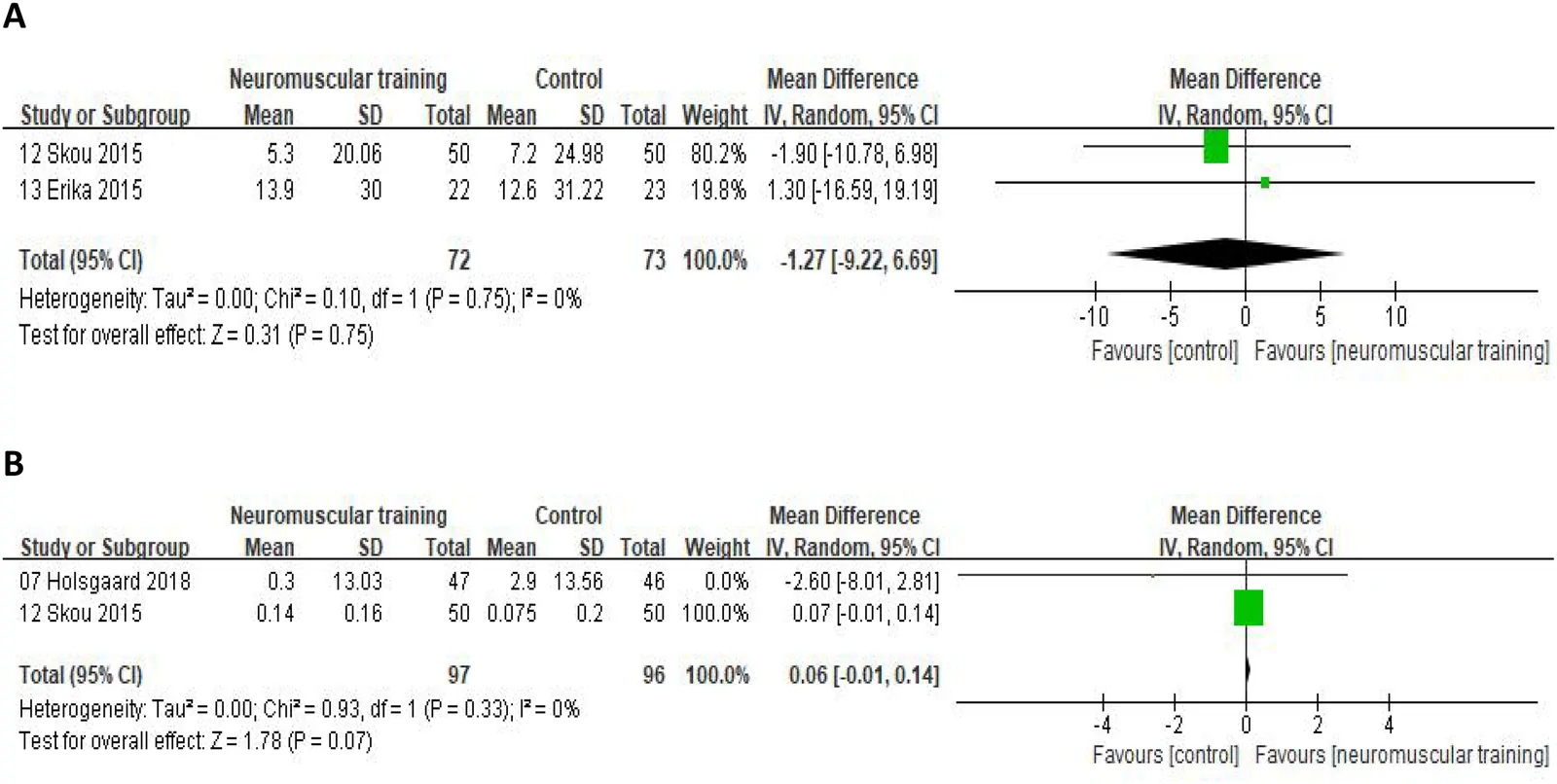
**(A)** Forest plot for the continuous variable EQ-5D-3L VAS. **(B)** Forest plot for the continuous variable EQ-5D-3L.

#### 3.6.3 Analysis of outcomes related to muscle and biomechanics

Among muscle strength-related indicators, isokinetic knee flexion peak torque, isokinetic knee extension peak torque, knee extensor strength (kg), and knee flexor strength (kg) all showed no statistically significant differences. Overall, the available evidence did not demonstrate a consistent improvement in muscle strength with NMT, and the number of studies for several biomechanical outcomes was limited. Further high-quality studies are needed to confirm these findings (**Figure 11**).

**Figure 11:**
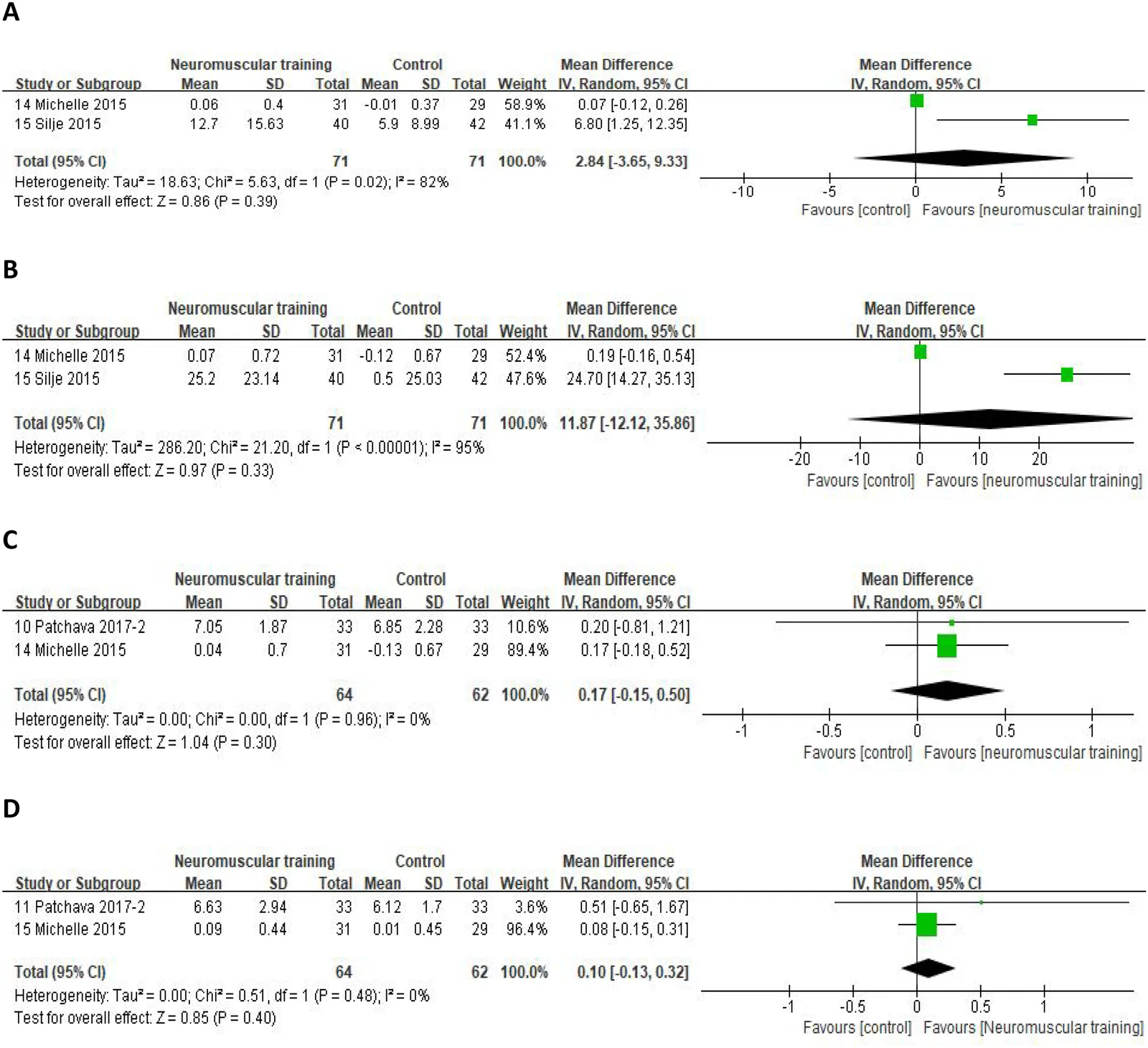
**(A)** Forest plot for the continuous variable Isokinetic knee flexion peak torque. **(B)** Forest plot for the continuous variable Isokinetic knee extension peak torque. **(C)** Forest plot for the continuous variable Knee Extensors (kg). **(D)** Forest plot for the continuous variable Knee Flexors (kg).

#### 3.6.4 Analysis of Western Ontario and McMaster Universities Osteoarthritis Index

Substantial between-study heterogeneity was observed among the three studies reporting WOMAC (*I*²=90%). Although all three effect estimates were in the direction favoring NMT, their magnitudes differed considerably. Sohrab 2023 reported a substantially larger between-group effect (MD = -27.27, 95% *CI*: -35.47, -19.07), whereas Joshi 2023 (MD = -3.82, 95% *CI*: -12.35, 4.71) and Shahzada 2019 (MD = −4.69, 95% *CI*: -13.66, 4.28) showed much smaller effects with confidence intervals crossing the null. This marked difference in effect magnitude, together with clinical and methodological differences across the studies, limited the interpretability of a single summary estimate. Therefore, quantitative pooling was not considered appropriate, and the WOMAC findings were synthesized narratively. Overall, the available evidence did not demonstrate a consistent magnitude of benefit of NMT on WOMAC scores.

#### 3.6.5 Analysis of outcomes related to functional performance

Most objective functional performance outcomes did not demonstrate statistically significant between-group differences, including the 30-Second Chair Stand Test, maximum number of knee bendings in 30 seconds, maximum number of one-leg rises from a stool, one-leg hop for distance, stair climb, Timed Up and Go and velocity of locomotion. However, NMT showed a statistically significant improvement in 20-m walk time (MD = -0.56, 95% *CI*: -1.10, -0.01, *P* = 0.04), although the clinical relevance of this difference remains uncertain. Overall, the available evidence suggests that NMT may provide potential benefits for certain aspects of objective functional performance; however, consistent evidence supporting broad improvements across functional outcomes remains insufficient (**Figure 12**).

**Figure 12:**
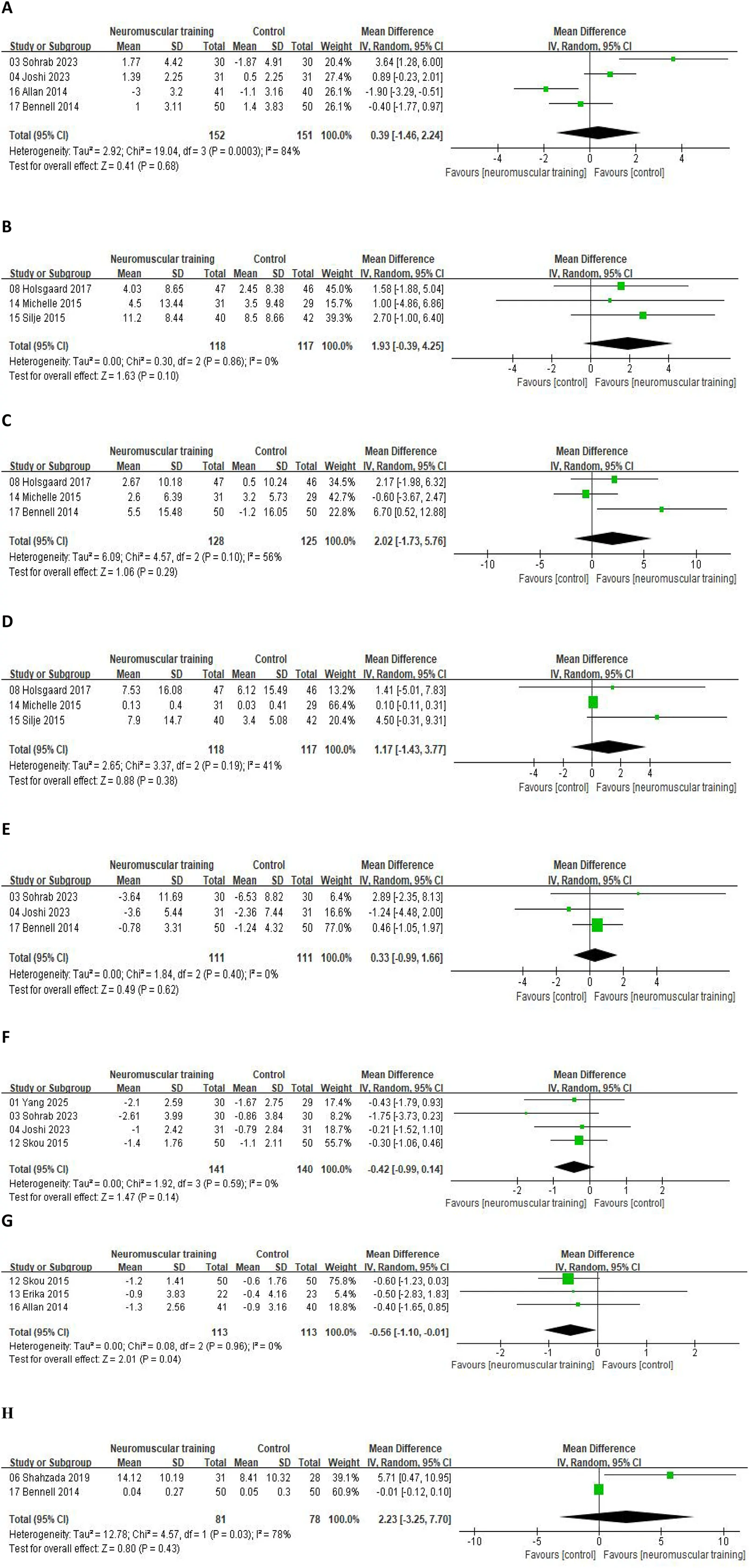
**(A)** Forest plot for the continuous variable 30-second chair stand test. **(B)** Forest plot for the continuous variable Maximum number of knee bendings in 30 seconds. **(C)** Forest plot for the continuous variable Maximum number of one-leg rises from a stool. **(D)** Forest plot for the continuous variable One-leg hop for distance. **(E)** Forest plot for the continuous variable Stair Climb. **(F)** Forest plot for the continuous variable Timed Up and Go. **(G)** Forest plot for the continuous variable 20-m walk (s). **(H)** Forest plot for the continuous variable Velocity of locomotion.

## 4 Discussion

This systematic review and meta-analysis found that the efficacy of NMT on KOOS Symptoms, KOOS ADLs, and KOOS QoL was statistically significant, while no differences were observed in its efficacy on KOOS Pain and KOOS Sport/Rec.

KOOS Symptoms encompasses stiffness, swelling, and other knee-related symptoms; therefore, its alleviation may reflect the potential impact of NMT on comprehensive symptoms. Theoretically, NMT may improve knee functional status and reduce mechanical discomfort by emphasizing motor control, periarticular muscle coordination, and dynamic stability. However, most objective biomechanical and functional indicators included in this study did not show statistically significant differences; accordingly, the above mechanisms remain primarily explanations at the level of biomechanical and neuromuscular control theory and cannot be directly confirmed by this meta-analysis. In the future, relevant studies need to incorporate objective indicators such as electromyography, joint loading, proprioception, and motor control to further verify the specific mechanisms by which NMT alleviates KOOS Symptoms.

NMT was statistically superior in improving KOOS QoL, suggesting that it may improve patients’ subjective assessment of QoL related to knee disease. KOOS QoL primarily reflects patients’ perceptions of the burden of knee disease, future function, and impact on daily life; therefore, this result suggests a potential benefit of NMT for knee-related QoL. This study revealed a statistically significant enhancement in KOOS QoL, which remained stable after sensitivity analysis. Many traditional meta-analyses tend to attach more importance to pain scores but underestimate the importance of QoL as a long-term rehabilitation goal. KOOS QoL reflects how patients perceive their disease burden, functional limitations, and confidence in future activities (Alshahrani et al., 2025). However, the observed mean difference between groups in this study was limited in magnitude, and statistical significance does not necessarily equate to clinically important improvement. Because all KOOS outcomes were analyzed on the original 0–100 scale, the pooled MDs directly represent differences in KOOS score units. The pooled effects for KOOS Symptoms, Pain, ADLs, Sport/Rec, and QoL were 4.44, 1.95, 3.94, 1.64, and 5.59 points, respectively, all of which were below the commonly cited 8-10-point threshold for a clinically important change in KOOS (Roos and Lohmander, 2003). However, because clinically important thresholds for KOOS are context-dependent and may vary according to the population and estimation method, these comparisons should be interpreted cautiously (Roos, 2024).

For KOA, a chronic, fluctuating, and long-term disease that profoundly affects lifestyle, improvement in KOOS QoL alleviates patients’ concerns about knee status and worries about future function, while enhancing self-perceived QoL. Wojcieszek et al. (2022) studied 300 subjects aged 60–75 years (150 patients with KOA and 150 controls) and found that greater KOA symptom severity, including pain, stiffness, and functional limitations, was associated with poorer overall QoL among older adults (*P* < 0.001). KOOS ADLs reflect the ability to perform ADLs, including stair climbing, standing up, walking, and completing basic life tasks (Roos, 2024). In the present meta-analysis, NMT was associated with a statistically significant improvement in KOOS ADLs compared with control interventions (MD = 3.94, 95% CI: 0.09, 7.79, *P* = 0.04). This finding suggests that the benefits of NMT may extend beyond symptom perception to basic functional performance in daily life. Improvement in KOOS ADLs may therefore indicate gains in patient-reported daily functional capacity beyond symptom relief alone. However, the pooled estimate was close to the threshold of statistical significance, the confidence interval was wide, and moderate heterogeneity was present (*I*²=54%). Therefore, although the current evidence supports a potential benefit of NMT for ADLs, the magnitude and clinical importance of this effect remain uncertain and require confirmation in larger, methodologically rigorous trials.

The absence of a statistically significant effect on KOOS Pain suggests that the benefits of NMT may not primarily operate through direct pain reduction. Pain is one of the most complex symptoms of KOA, influenced by multiple factors (e.g., peripheral inflammation, structural changes in cartilage and subchondral bone, central sensitization, emotional state, sleep quality, and past pain memory) (Uivaraseanu et al., 2022). NMT influences pain indirectly by optimizing motor control, reducing functional instability, and reducing joint loads, rather than directly acting on pain pathways as drugs do. Consequently, no significant advantage was observed in pain reduction. Despite the non-significant effect on KOOS Pain, significant improvements were observed in KOOS Symptoms and KOOS QoL, indicating that the pattern of patient benefit is not ‘simple pain relief’, but rather overall alleviation in symptom perception and enhancement in QoL.

The lack of a significant effect on KOOS Sport/Rec may reflect the greater physical demands captured by this domain. KOOS Sport/Rec imposes higher demands on the knee joint, requiring not only reduced pain and enhanced basic activity ability, but also greater explosive strength, endurance, speed, coordination, psychological confidence, and a firm willingness to participate in higher-level activities (Roos and Lohmander, 2003). In patients with KOA, who are often older and may have obesity or long-standing functional limitations, improvements in symptoms and QoL may not translate into short-term gains in sport and recreational function. Therefore, the non-significant effect on KOOS Sport/Rec is not contradictory to the positive effects observed in other domains.

Therefore, one possible explanation is that the intensity, duration, or goal setting of the current NMT regimens may be insufficient to produce detectable improvements in high-level functional capacity. However, given the limited number of included studies and the fact that most objective functional outcomes also did not show consistent advantages, it remains uncertain whether this negative result is due to the limited efficacy of NMT itself, or to insufficient statistical power, differences in training dosage, or patient characteristics.

In this study, we further conducted pre-specified subgroup analyses according to control type, intervention duration, mean age, BMI, and geographic region. Statistically significant effects were observed within some subgroups. The results showed that KOOS Symptoms were statistically alleviated in the ‘rehabilitation exercise alone’ subgroup, the ‘pharmacotherapy alone’ subgroup, the ‘mean age ≥60 years’ subgroup, the ‘mean age <60 years’ subgroup, the ‘BMI ≥28 kg/m²’ subgroup, and the ‘Europe’ subgroup. Statistical differences in the mitigation of KOOS Pain were observed only in the ‘Europe’ subgroup. Statistical differences in increasing KOOS ADLs were noted only in the ‘mean age ≥60 years’ subgroup. Statistical differences in improving KOOS QoL were found in the ‘mean age ≥60 years’ subgroup and the ‘BMI ≥28 kg/m²’ subgroup. In contrast, no statistically significant differences were observed in improving KOOS Sport/Rec in the overall analysis or in any pre-specified subgroups. These results suggest that under certain clinical and study conditions, the effect of NMT may be more readily observed. However, interaction tests between subgroups did not provide sufficient evidence to support that control type, intervention duration, BMI, or geographic region constitute definite effect modifiers. A statistically significant subgroup interaction was observed for mean age in the KOOS QoL analysis (*P* for interaction = 0.02), suggesting a potential age-related difference in the effect of NMT on knee-related QoL. Nevertheless, given the limited number of studies within the age subgroups and the use of study-level mean age rather than individual participant data, this finding should be interpreted cautiously and regarded as exploratory. The findings related to BMI and geographic region should likewise not be interpreted as evidence of definitive effect modification. In the future, pre-specified stratified RCTs with adequate sample sizes or individual participant data meta-analyses are warranted to further validate these findings.

The overall pattern of the secondary outcomes further supports this interpretation. No statistically significant differences were observed in most muscle strength outcomes, pain-related measures, general health status measures, gait parameters, physical performance tests, or biomechanical indicators. For a chronic degenerative disease such as KOA, the benefits of NMT appear to be more evident in symptom control and QoL, with limited and outcome-specific evidence of improvement in objective functional performance. These effects may not be fully captured by individual functional tests or biomechanical parameters over relatively short follow-up periods. In clinical practice, NMT may therefore be considered as one component of a comprehensive rehabilitation strategy rather than a standalone intervention addressing all clinical aspects of KOA.

Compared with previous meta-analyses, this study offers several notable advances. Emami et al. (2026) included only eight clinical trials with a total of 608 participants and analyzed only outcomes such as KOOS, WOMAC, Stair Climb test, Chair Stand test, Y Balance test, and Timed Up and Go test. Their results demonstrated a significant improvement in KOOS QoL favoring NMT, but no clear statistical advantage was observed in other domains. Moreover, the study by Emami et al. revealed that the limited number of included studies precluded formal subgroup analyses to further explore the sources of heterogeneity. In contrast, this study included 17 study reports from 14 unique RCTs involving 1,029 randomized participants and evaluated a broader range of patient-reported and objective outcomes. Rather than restricting itself to overall functional measures, this study constructed a more comprehensive outcome assessment system by systematically analyzing the five KOOS dimensions (Symptoms, Pain, ADLs, Sport/Rec, and QoL). In addition, we incorporated adverse events, pain-related secondary outcomes, general HRQoL, WOMAC, muscle strength indicators, biomechanical parameters, and multiple objective functional performance measures. Consequently, this study more comprehensively reflects the combined effects of NMT on symptoms, pain, ADLs, QoL, and safety in individuals with KOA. Moreover, in this study, sensitivity analyses, publication bias assessments, and subgroup analyses stratified by control type, intervention duration, mean age, mean BMI, and study location were further performed to explore potential effect modifiers and sources of heterogeneity. These measures addressed a key limitation of previous studies, in which formal subgroup analyses were not feasible owing to the small number of included trials. Thus, this study may provide a more detailed, comprehensive, and practically valuable evidence base for clinical rehabilitation decision-making than previously available.

Several limitations of this study should be acknowledged. First, some heterogeneity existed across included studies in terms of NMT intervention regimens, including training content, frequency, session duration, total intervention period, supervision method, adherence management for home-based training, and whether combined with pharmacotherapy, health education, or other rehabilitation measures. These variations may weaken the stability and comparability of the pooled intervention effects. Although we adopted a random-effects model and conducted sensitivity and subgroup analyses, clinical heterogeneity may not have been fully eliminated. Moderate to high heterogeneity was observed for KOOS ADLs (*I²* = 54%) and KOOS QoL (*I²* = 67%). The prespecified subgroup variables did not fully account for the observed between-study heterogeneity; although mean age showed a statistically significant interaction for KOOS QoL (*P* for interaction = 0.02), this finding was exploratory and did not fully explain the observed heterogeneity. Second, the number of included studies for several secondary outcomes was relatively small, limiting statistical power. Third, while Egger’s test revealed no significant publication bias for most outcomes, the small number of studies in some analyses meant that negative results could not completely rule out bias.

Future studies should focus on standardized NMT protocols, objective monitoring of adherence and training dose, and adequately powered RCTs with longer follow-up. Wearable sensors and remote monitoring may facilitate more precise assessment of exercise exposure and functional response (Xiang et al., 2024; Xie et al., 2024). Concurrently, additional research into the mechanisms of psychological factor involvement may help to construct a more complete rehabilitation system and promote holistic physical and mental health. NMT is a significant component of rehabilitation for patients with KOA. Clinical NMT practice in the future should emphasize multimodal comprehensive interventions and individualized regimen design, integrating scientific evidence with clinical experience, to advance the field of KOA rehabilitation and ultimately achieve the goals of enhancing patients’ QoL and functional status.

## 5 Conclusion

NMT may partially alleviate comprehensive symptoms, facilitate activities of daily living, and enhance knee-related QoL in patients with KOA, with no statistically significant difference in reported adverse events. Nevertheless, consistent evidence remains lacking regarding the benefits of NMT in pain relief, sport and recreational function, and most objective functional performance indicators. In the future, additional large-scale, high-quality RCTs with standardized intervention regimens are warranted to further validate the true effects of NMT, identify the patient subgroups most likely to benefit, and determine its long-term clinical value.
