# Supplementary material for: Rehabilitation efficacy of neuromuscular training for individuals suffering from knee osteoarthritis: a systematic review and meta-analysis based on randomized controlled trials
Source: Front Physiol. 2026 Oct 21;17:1892963. doi: 10.3389/fphys.2026.1892963 (PMC13730944; doi:10.3389/fphys.2026.1892963)
Supplement: Supplementary file 1 [file Table1.docx]

Supplementary Table S1.the literature search strategy

Pubmed-166

(((neuromuscular) AND (((training) OR (exercise)) OR (sport))) AND (("Osteoarthritis, Knee"[Mesh]) OR ((((Knee Osteoarthritides) OR (Knee Osteoarthritis)) OR (Osteoarthritis of Knee)) OR (Osteoarthritis of the Knee)))) AND (Random*)

Embase-281

(((neuromuscular) AND (((training) OR (exercise)) OR (sport))) AND ((Osteoarthritis, Knee) OR ((((Knee Osteoarthritides) OR (Knee Osteoarthritis)) OR (Osteoarthritis of Knee)) OR (Osteoarthritis of the Knee)))) AND (Random*)

Cochrane-222

(((neuromuscular) AND (((training) OR (exercise)) OR (sport))) AND ((Osteoarthritis, Knee) OR ((((Knee Osteoarthritides) OR (Knee Osteoarthritis)) OR (Osteoarthritis of Knee)) OR (Osteoarthritis of the Knee)))) AND (Random*)

Web of Science-223

(((neuromuscular) AND (((training) OR (exercise)) OR (sport))) AND ((Osteoarthritis, Knee) OR ((((Knee Osteoarthritides) OR (Knee Osteoarthritis)) OR (Osteoarthritis of Knee)) OR (Osteoarthritis of the Knee)))) AND (Random*) (Topic)
