# Supplementary material for: Rehabilitation efficacy of neuromuscular training for individuals suffering from knee osteoarthritis: a systematic review and meta-analysis based on randomized controlled trials
Source: Front Physiol. 2026 Oct 21;17:1892963. doi: 10.3389/fphys.2026.1892963 (PMC13730944; doi:10.3389/fphys.2026.1892963)
Supplement: Supplementary file 2 [file Table3.docx]

**Supplementary Table 3. Trial-family mapping of multiple reports originating from the same randomized trial**

| **Trial family** | **Publication/report** | **Trial registration** | **Randomized sample** | **Intervention / control** | **Follow-up represented** | **Main outcomes contributed** | **Relationship between reports** | **Handling in this review** |
| --- | --- | --- | --- | --- | --- | --- | --- | --- |
| EXERPHARMA | Holsgaard 2017 | NCT01638962 | NEMEX 47 / PHARMA 46 | NEMEX / instruction in analgesic use | 2 months | KOOS Symptoms, Pain, ADL, Sport/Rec, QoL; biomechanical outcomes | Primary/short-term report of the EXERPHARMA trial | Classified within the same trial family as Holsgaard 2018; short-term data used where applicable; not counted as an additional trial. |
| EXERPHARMA | Holsgaard 2018 | NCT01638962 | NEMEX 47 / PHARMA 46 | NEMEX / instruction in analgesic use | 12 months | KOOS and other patient-reported outcomes | Extended 12-month follow-up of the same EXERPHARMA randomized cohort | Retained as a follow-up report and source of long-term information; not counted as an additional independent RCT. |
| Rashid trial | Shahzada 2019 | Not clearly reported in the available report | NMT 33 / QT 33 | NMT / quadriceps training | Post-intervention | WOMAC, gait-related outcomes, adverse-event information where reported | Primary clinical report of the randomized cohort | Treated as the primary report of this trial family; randomized participants counted once at the trial level. |
| Rashid trial | Shahzada 2022 | Not clearly reported in the available report | Same underlying randomized cohort (33 / 33 randomized) | NMT / quadriceps training | Post-intervention | Muscle activation/EMG and co-contraction index | Secondary analysis of the randomized clinical trial reported in the primary paper | Used for complementary neuromuscular outcomes; not counted as an additional independent RCT or an additional randomized cohort. |
| MEDIC trial | Skou 2015 | NCT01535001 | MEDIC 50 / usual care 50 | MEDIC multimodal non-surgical treatment including NEMEX / usual care | 12 weeks and 12 months | KOOS, functional outcomes, adverse events | Main RCT report | Treated as the primary report of the MEDIC trial; randomized participants counted once at the trial level. |
| MEDIC trial | Skou 2016 | NCT01535001 | MEDIC 50 / usual care 50 | Same randomized interventions as Skou 2015 | 3 months | Pain intensity, pain sensitization and pressure pain thresholds | Pre-defined ancillary analysis of the same MEDIC randomized trial | Used for complementary pain/sensitization outcomes; not counted as an additional independent RCT. |

**Note:** Publications within the same trial family originated from the same underlying randomized cohort and therefore were not considered independent randomized controlled trials. Multiple reports were retained when they provided complementary outcomes, secondary analyses, or different follow-up assessments. The number of unique trials and randomized participants should be calculated at the underlying trial level rather than the publication level.
