# Supplementary material for: Rehabilitation efficacy of neuromuscular training for individuals suffering from knee osteoarthritis: a systematic review and meta-analysis based on randomized controlled trials
Source: Front Physiol. 2026 Oct 21;17:1892963. doi: 10.3389/fphys.2026.1892963 (PMC13730944; doi:10.3389/fphys.2026.1892963)
Supplement: Supplementary file 3 [file Table4.docx]

**Supplementary Table S4.The quality of the eligible studies was assessed according to the Cochrane Handbook for Systematic Reviews of Interventions, Version 5.1.0.**

| **Study** | | **Random sequence generation** | **Allocation concealment** | **Blinding** | | **Incomplete outcome data** | **Selective reporting** | **Other sources of bias** |
| --- | --- | --- | --- | --- | --- | --- | --- | --- |
|  |  |  |  | **Blinding of participants and personnel** | **Blinding of outcome assessment** |  |  |  |
| 1 | **Yang 2025** | Low risk | Low risk | Low risk | Low risk | Low risk | Low risk | Low risk |
| 2 | **Lisa 2025** | Low risk | Low risk | Low risk | Low risk | Low risk | Low risk | Low risk |
| 3 | **Sohrab2023** | Low risk | Low risk | High risk | Unclear | Low risk | Unclear | Low risk |
| 4 | **Joshi 2023** | Low risk | Low risk | High risk | Unclear | Low risk | Low risk | Low risk |
| 5 | **Shahzada 2022** | High risk | High risk | High risk | Low risk | Low risk | Unclear | Low risk |
| 6 | **Shahzada 2019** | Low risk | High risk | High risk | Unclear | Low risk | Unclear | Low risk |
| 7 | **Holsgaard 2018** | Low risk | Low risk | High risk | Low risk | Low risk | Low risk | Low risk |
| 8 | **Holsgaard 2017** | Low risk | Low risk | High risk | Low risk | Low risk | Low risk | Low risk |
| 9 | **Patchava 2017-1** | Low risk | Low risk | High risk | Unclear | Low risk | Unclear | Low risk |
| 10 | **Patchava 2017-2** | Low risk | High risk | High risk | Unclear | Low risk | Unclear | Low risk |
| 11 | **Skou 2016** | Low risk | Low risk | Low risk | Low risk | Low risk | Low risk | Low risk |
| 12 | **Skou 2015** | Low risk | Low risk | High risk | Low risk | Low risk | Low risk | Low risk |
| 13 | **Erika 2015** | Low risk | Low risk | High risk | Low risk | Low risk | Low risk | Low risk |
| 14 | **Michelle 2015** | Low risk | Low risk | High risk | Low risk | Low risk | Low risk | Low risk |
| 15 | **Silje 2015** | Low risk | Low risk | High risk | Unclear | Low risk | Low risk | Low risk |
| 16 | **Allan 2014** | Low risk | Low risk | High risk | Low risk | Low risk | Low risk | Low risk |
| 17 | **Bennell 2014** | Low risk | Low risk | High risk | Low risk | Low risk | Low risk | Low risk |
