# Supplementary material for: Rehabilitation efficacy of neuromuscular training for individuals suffering from knee osteoarthritis: a systematic review and meta-analysis based on randomized controlled trials
Source: Front Physiol. 2026 Oct 21;17:1892963. doi: 10.3389/fphys.2026.1892963 (PMC13730944; doi:10.3389/fphys.2026.1892963)
Supplement: Supplementary file 4 [file Table5.docx]

| Supplementary Table S5. GRADE rating of each outcome. | | | | | | | | | | | | | |
| --- | --- | --- | --- | --- | --- | --- | --- | --- | --- | --- | --- | --- | --- |
| No. of RCTs | Outcomes | MD/RR | 95% CI | I*^2^*; *P value for heterogeneity* | Risk of bias | Inconsistency | Indirectness | Imprecision | Publication bias | Plausible confounding | Magnitude of effect | Dose-response gradient | GRADE |
| 8 | KOOS Symptoms | 4.44 | 1.62, 7.26 | 23%; *P*=0.24 | Serious risk | No serious inconsistency | No serious  indirectness | No serious imprecision | NA | Would not reduce effect | No | No | Moderate |
| 9 | KOOS Pain | 1.95 | -0.27, 4.17 | 0%; *P*=0.56 | Serious risk | No serious inconsistency | No serious  indirectness | Serious imprecision | Undetected | Would not reduce effect | No | No | Low |
| 7 | KOOS ADL | 3.94 | 0.09, 7.79 | 54%; *P*=0.04 | Serious risk | Serious inconsistency | No serious  indirectness | Serious imprecision | NA | Would not reduce effect | No | No | Very low |
| 6 | KOOS Sport/Rec | 1.64 | -2.24, 5.53 | 0%; *P*=0.93 | Serious risk | No serious inconsistency | No serious  indirectness | Serious imprecision | NA | Would not reduce effect | No | No | Low |
| 9 | KOOS QoL | 5.59 | 0.97,10.21 | 71%; *P*=0.001 | Serious risk | Serious inconsistency | No serious  indirectness | No serious imprecision | Undetected | Would not reduce effect | No | No | Low |
| 12 | Adverse events | 0.83 | 0.50, 1.37 | 0%; *P*=0.62 | Serious risk | No serious inconsistency | No serious  indirectness | Serious imprecision | Strongly  suspected | Would not reduce effect | No | No | Very low |

Notes: MD, mean difference; RR, risk ratio; 95% CI, corresponding 95% confidence interval; RCT, randomized controlled trial; NA, not assessed/applicable.
